# Sophistication in a seemingly simple creature: a review of wild holothurian nutrition in marine ecosystems

**DOI:** 10.1111/brv.12799

**Published:** 2021-10-13

**Authors:** Joséphine Pierrat, Alexandre Bédier, Igor Eeckhaut, Hélène Magalon, Patrick Frouin

**Affiliations:** ^1^ UMR ENTROPIE (IRD, CNRS, Univ. Reunion, Ifremer, Univ. New Caledonia) University of La Réunion St‐Denis 97 400 France; ^2^ Nutrima Production Le Port 97 420 France; ^3^ Biology of Marine Organisms and Biomimetism Lab University of Mons Mons 7000 Belgium; ^4^ Labex Corail Perpignan 66 000 France

**Keywords:** holothurians, tentacle ultrastructure, bud epidermis, mucous cell, trophic mode, selective feeding, dietary, proteobacteria, plastic particles

## Abstract

Holothurians are marine invertebrates that are among the most widespread benthic megafauna communities by both biomass and abundance in shallow‐water and deep‐sea ecosystems, their functions supporting important ecological services worldwide. Despite their simple appearance as sea cucumbers, holothurians show a wide range of feeding practices. However, information on what and how these animals eat is scattered and potentially confusing. We provide a comprehensive review of holothurian nutrition in coastal and deep‐sea ecosystems. First, we describe morphological aspects of holothurian feeding and the ultrastructure of tentacles. We discuss the two processes for food capture, concluding that mucus adhesion is likely the main method; two mucous cells, type‐1 and type‐2, possibly allow the adhesion and de‐adhesion, respectively, of food particles. Secondly, this review aims to clarify behavioural aspects of holothurian suspension‐ and deposit‐feeding. We discuss the daily feeding cycle, and selective feeding strategies. We conclude that there is selectivity for fine and organically rich particles, and that feeding through the cloaca is also a route for nutrient absorption. Third, we provide a wide description of the diet of holothurians, which can be split into two categories: living and non‐living material. We suggest that Synallactida, Molpadida, Persiculida, Holothuriida and Elasipodida, ingest the same fractions, and emphasise the importance of bacteria in the diet of holothurians.

## I. INTRODUCTION

Dating back 460 million years, holothurians (commonly called sea cucumbers) are ubiquitous marine echinoderms belonging to the class Holothuroidea. An initial classification was established by Pearson (1914), who attempted to organise the species, under the name *Holothuria* L., into groups based on their gross morphology. Pawson & Fell (1965) subsequently proposed a classification based on tentacle, body, and calcareous ring morphology, defining five orders: Dendrochirotida, Apodida, Molpadida, Elasipodida, and Aspidochirotida. Until recently, this was the most widely used classification for holothurians. When the first broad‐scale molecular phylogenetic analyses of Holothuroidea were completed, these deeply altered the previous classification through the suppression of the order Aspidochirotida, which was revealed to be polyphyletic. Species previously under Aspidochirotida have since been placed into three new orders: Synallactida, Persiculida (in part), and Holothuriida (Miller *et al*., 2017). There are more than 1,752 accepted holothurian species (WoRMS, 2020), with new species being described each year.

Holothurians have a global distribution, colonising all biotopes of the ocean from the polar front (Lawrence & Guille, 1982; Féral & Magniez, 1985; Gutt, 1990; Post *et al*., 2017; O'Loughlin, Bardsley & O'Hara, 2020) to the tropical zone (Sloan & von Bodungen, 1980; Wiedemeyer, 1994; Asha *et al*., 2015; Resueño & Angara, 2020), with most species inhabiting the tropical Indo‐West Pacific region (Conand, 1990). They have also colonised all depths, from shallow‐water (Jaquemet, Rousset & Conand, 1999; Dissanayake & Stefansson, 2010; MacTavish *et al*., 2012; Lee *et al*., 2017) to hadal zones (Iken *et al*., 2001; Jamieson *et al*., 2011). They are among the most widespread benthic megafauna species in terms of biomass and abundance in many ecosystems, especially in the hadal zone, considered ‘the kingdom of Holothuroidea’ (Beliaev & Brueggeman, 1989; Kuhnz *et al*., 2014), in coral reefs and lagoons (Uthicke, 1999; Wolfe & Davey, 2020), and in sheltered marine shallow habitats (Conde, Diaz & Sambrani, 1991).

Some species of holothurians are considered as luxury food (bêche‐de‐mer, trepang or hai‐som), medicines, and aphrodisiacs in many Asian countries (Conand, 1990; Lovatelli *et al*., 2004; Shiell & Uthicke, 2005; Toral‐Granda, Lovatelli & Vasconcellos, 2008). More than 70 species of holothurians are commonly harvested (Purcell *et al*., 2016), predominantly from the Indo‐Pacific region (Kinch *et al*., 2008; Conand, 2018). In some locations, populations of highly commercially valuable species have been decimated to a point that fishing regulations and regulatory measures alone may be insufficient to restore populations (Friedman *et al*., 2011). This overexploitation is linked to a shift from traditional to semi‐industrial fisheries (Conand, 2001). Countries of the Indian Ocean, West Pacific and Latin America have active fisheries (Conand, 2018), and South‐East Asia is considered the main world market (Rahman & Yusoff, 2017). The world fishery of holothurians quadrupled between 1955 and 2012 to satisfy the increasing Asian market for ‘bêche‐de‐mer’ (Rahman, Yusoff & Arshad, 2015). Consequently, many countries in the Indo‐Pacific have prioritised sea cucumber aquaculture in their development plans (Jimmy, Pickering & Hair, 2012). In addition, restocking, sea ranching, and sea farming have been described as potential alternatives to reduce pressure on wild holothurian populations and their worldwide overexploitation. Three species have been registered recently in CITES (Convention on International Trade in Endangered Species of Wild Fauna and Flora) Appendix II: *Holothuria* (*Microthele*) *fuscogilva* Cherbonnier, *Holothuria* (*Microthele*) *nobilis* (Selenka), and *Holothuria* (*Microthele*) *whitmaei* Bell, each with high commercial value but declining natural stocks (Di Simone, Horellou & Conand, 2019).

**Fig 1:**
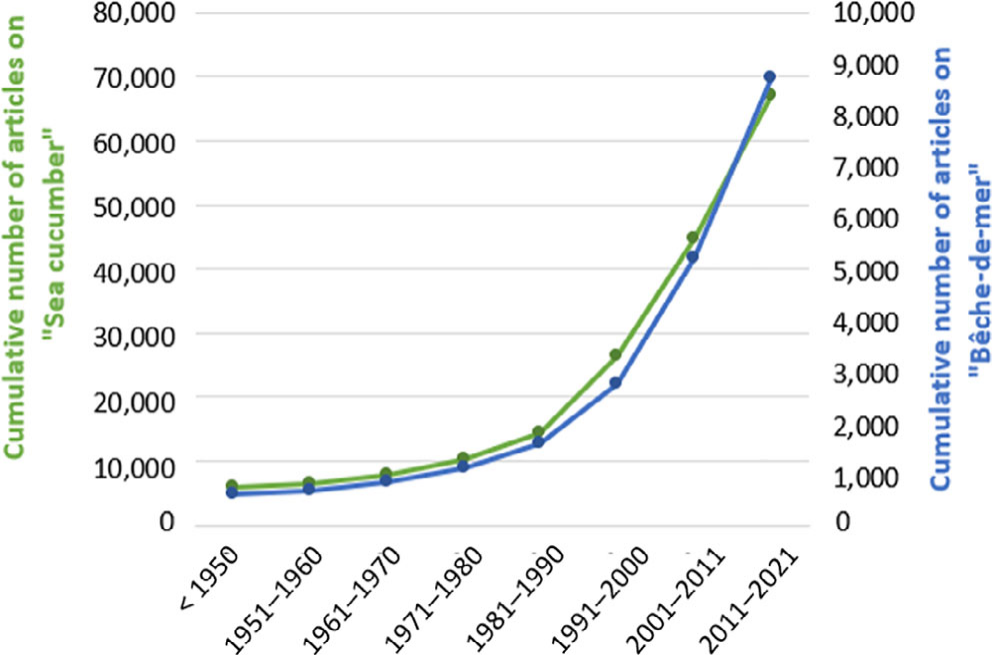
Cumulative number of articles on “sea cucumber” for general publications and “bêche‐de‐mer” for those focusing on fisheries and aquaculture since 1950. Note the different scales for the two axes.

Due to their global distribution favouring many local studies, and their importance in Asian culture, holothurians have gained increasing importance in recent decades. A search on *Google Scholar* using the key words ‘sea cucumber’ for general publications and ‘bêche‐de‐mer’ for those focusing on fisheries and aquaculture, shows that the number of articles on both topics has increased considerably since the 1990s (Fig. 1).

The increasing literature provides scattered data about holothurian feeding, making it difficult to obtain a broad view on this topic. This review intends to assess our understanding of holothurian feeding. The nutritional aspects considered herein include feeding (the act of consuming food), digestion and absorption. To our knowledge, only one review has been published previously on the nutrition of holothurians (edited by Jangoux & Lawrence, 1982), about 40 years ago. There is no comprehensive review covering the nutrition traits of holothurians in relation to their ecology, and collating information on their feeding patterns. While much information exists on the feeding of farmed holothurians, this relates only to controlled conditions. Thus, our aim herein is to analyse holistically the nutrition of wild holothurians.

## II. MORPHOLOGICAL AND PHYSIOLOGICAL ASPECTS

### (1) Tentacle structure and movements during ingestion

The number, size, and structure of tentacles varies among holothurian orders. Based on their structure, Massin (1982) defined five types of tentacles: dendritic, peltate, pinnate, digitate, and peltodendritic. The first four of these are common and widely used in the description of tentacle morphology of holothurians. Species within Dendrochirotida possess dendritic tentacles whereas those of Molpadida possess digitate tentacles (Levin, 1989). Peltate tentacles, described as “cauliflower‐like structures” (Bouland, Massin & Jangoux, 1982, p. 134) or “a nasturtium leaf with a central short stalk giving off horizontal branches” (Hyman, 1955, *cf*. Cameron & Fankboner, 1984, p. 193), are found within Elasipodida, Synallactida, Persiculida, and Holothuriida. In some cases, the tentacle structure shows variations among species within the same order, such as for Apodida species, which are found in coastal and deep‐sea ecosystems and possess pinnate or digitate tentacles. Combinations of these four main types of tentacle can be found in the literature: (*i*) a ‘peltodendritic tentacle’ which is, according to Massin (1982), a combination of the shaft of a peltate tentacle and distal end of a dendritic tentacle; (*ii*) a ‘peltatodigitate tentacle’, which is a combination of the shaft of a peltate tentacle and distal end of a digitate tentacle (Miller *et al*., 2017). Peltodentritic tentacles are uncommon, while peltatodigitate tentacles are found in Apodida (Miller *et al*., 2017).

**Fig 2:**
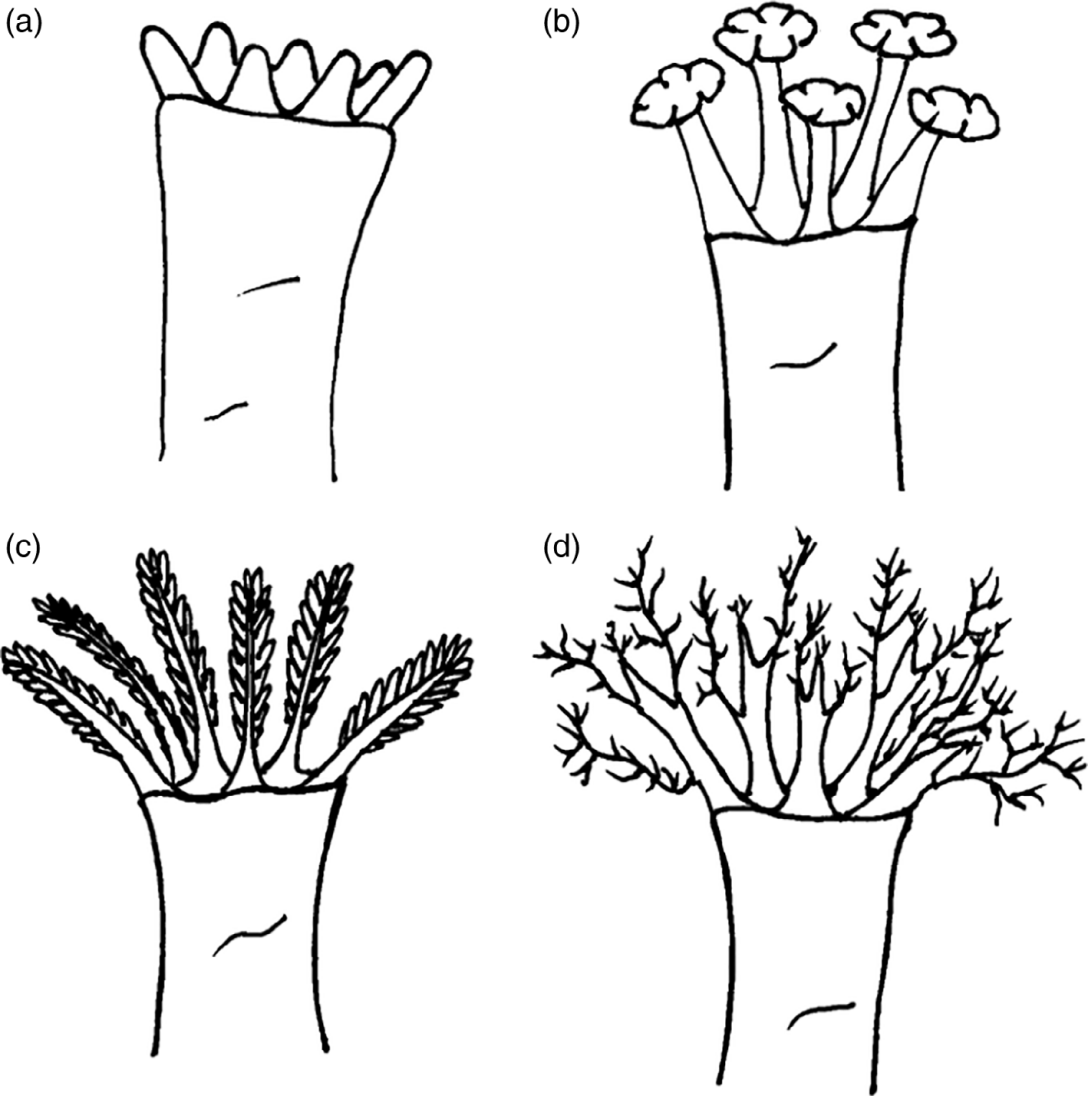
Examples of holothurian tentacle morphology. (A) Digitate (unbranched); (B) peltate (slightly branched); (C) pinnate (highly branched); (D) dendritic (ultra‐branched).

**Fig 3:**
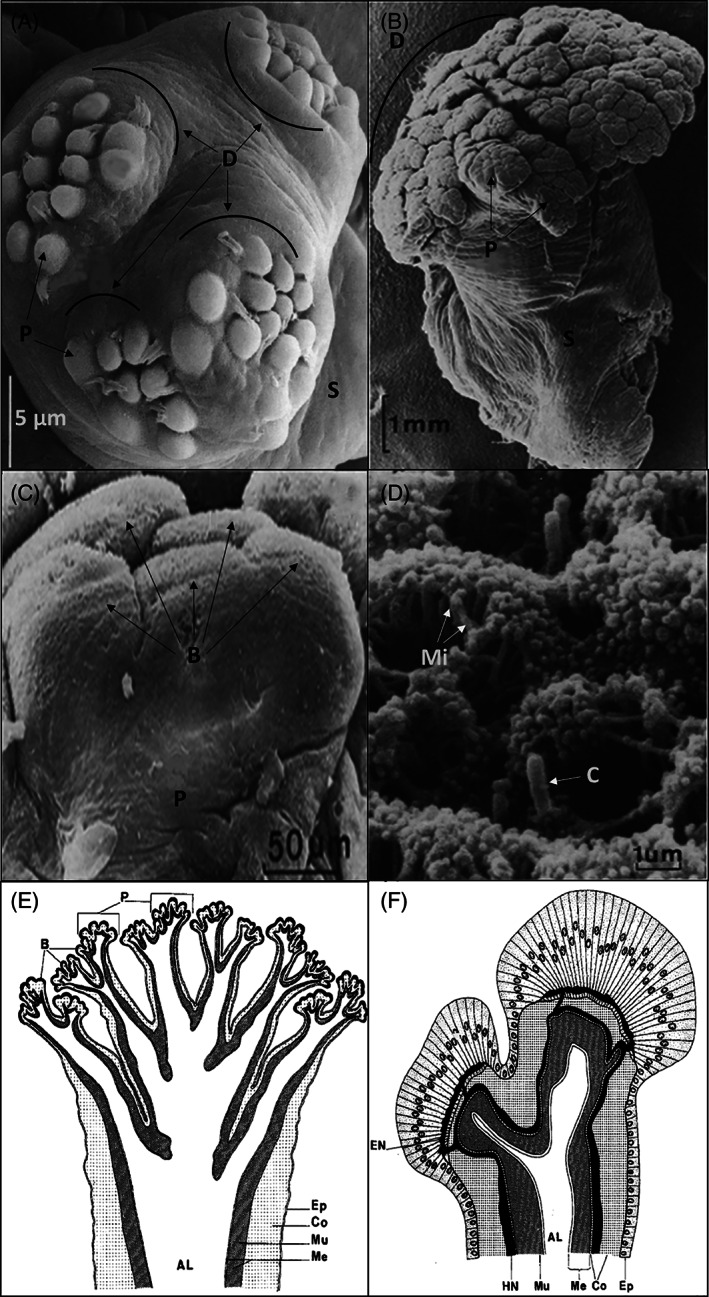
Fine ultrastructure of the holothurian tentacle. Digitate tentacle of (A) *Thyonidium* sp. and (B) *Holothuria forskali*. D, discs; P, Papillae; S, shaft. (C) Profile view of a five‐bud papilla. B, buds. (D) Arrangement of cilia and microvilli on bud surface. C, cilium; Mi, microvilli. (E, F) Diagrammatic sections through a whole tentacle (E) and through a two‐bud papilla (F). AL, ambulacral lumen; Co, connective tissue layer; EN, epineural nerve plexus; Ep, epidermis; HN, hyponeural nerve plexus; Me, mesothelium; Mu, muscles. Modified from Bouland *et al*. (1982) and McKenzie (1987).

Tentacle morphology may also differ between early and adult stages of the same species (Cameron & Fankboner, 1984). Generally, a tentacle possesses several shafts that end with one or more discs, composed of numerous apical papillae (Fig. 3A, B). The apex of each papilla is characterised by the presence of buds (Fig. 3C) with various cell types. The main difference in tentacle structure is the degree of branching of the main tentacle stalk (Fig. 2), from an unbranched (digitate form), to a slightly branched (peltate form), to a highly branched (pinnate form) to an ultra‐branched (dendritic form) structure. These differences reveal an adaptive radiation facilitating habitat and feeding specialisations (Sokolova, 1958; Hansen, 1975). From an ecological perspective, differences in tentacular morphology among sympatric holothurian species with overlapping bathymetric ranges may allow resource partitioning to avoid competitive interactions (Roberts & Moore, 1997).

A comprehensive review by Massin (1982) described the feeding mechanisms of deposit‐feeding holothurians. When the animal initiates feeding, the tentacles expand into the water column, and are pressed onto or into the sediment surface where they perform investigatory movements (Bouland *et al*., 1982). The tentacular movement is thought to be driven by the hydrostatic pressure of the ambulacral fluid of the water‐vascular system and the mesothelial muscles (Bouland *et al*., 1982). Tentacles can also cooperate to capture large debris such as fragments of seagrass or macroalgae. In this case, when the desired food elements are located, the extremities of the tentacle retract first, thus entrapping the particles between the buds (Fankboner, 1978; Levin, 1989). Sokolova (1958) described how tentacle morphology can influence the collection of particulate food in deep‐sea holothurian species; well‐developed marginal processes on discs are capable of picking up single food particles from the sediment, whereas species with undifferentiated discs indiscriminately ingest the upper layer of the sediment. After collection, the tentacle continues to contract, bending towards the mouth and penetrating it. At this ingestion stage, mouth size is the limiting factor (Myers, 1977), although a proportion of captured particles is lost during transport to the mouth (Powell, 1977; Levin, 1989). As the tentacle penetrates the pharyngeal cavity, particle removal is facilitated by wiping the tentacle against the bulging pharynx wall (Cameron & Fankboner, 1984). Finally, the tentacle withdraws from the mouth and extends again to continue investigating for food sources.

### (2) Food‐capture mechanism

Understanding the mechanism(s) of capture of food particles in holothurians was approached *via* studies in functional morphology integrating data concerning the ultrastructure of the tentacles to determine the composition of the tentacle parts in contact with the ingested sediment. These studies, mainly carried out in the 1980s and 1990s, are unfortunately few in number. Sixteen species were investigated, mainly Dendrochirotida (13 species; Smith, 1983; McKenzie, 1987), two former Aspirochirotida (Bouland *et al*., 1982; Cameron & Fankboner, 1984) and one Apodida (Flammang & Conand, 2004). Roberts & Moore (1997) and Fankboner (1981) used scanning electron microscopy to study the fine external structure of tentacles of four species of Elasipodida and five species of Dendrochirotida without detailing their cellular composition. Based on these studies and behavioural experiments, several authors have proposed mechanisms for the capture of food particles in several species (Roberts, 1979; Hammond, 1982; Bouland *et al*., 1982; Cameron & Fankboner, 1984). Holothurians appear to use two methods for food collection: food particle ensnarement (a mechanical process) and food particle adhesion (a chemical process). Food particle ensnarement was proposed in early studies about the functioning of the tentacles, since when adhesion has gained support as the principal mechanism involved in the capture of food particles.

#### (a) Food particle adhesion

Several authors described a mucus‐like secretion on the oral tentacles and suggested a primary role in holothurian food capture (Bouland *et al*., 1982). Roberts & Bryce (1982) described mucus‐secretory cells in the tentacular epidermis of several tropical species and stated that the adhesive material would play a role in collecting food particles. Levin (1982), studying *Apostichopus japonicus* (Selenka), presumed that adhesion was a primary function of the tentacle during feeding. Similarly, Hammond (1982) observed mucus coating the tentacular surface and stated that adhesion is a significant factor in food collection for Synallactida, Persiculida, and Holothuriida, a statement reiterated by other authors working on temperate species (Smith, 1983; Costelloe & Keegan, 1984; Holtz & MacDonald, 2009).

Transmission electron microscopy studies have shown buds to be the sites of secretion. The bud epidermis can include six types of cells in the species investigated to date, with 3–5 of these cell types usually being present (Table 1). The functions of these cells have been deduced by drawing parallels with the roles of cells in the adhesive disc of podia of echinoderms. (*i*) ‘Support cells’ have been described in some Dendrochirotida (McKenzie, 1987) and in the only species of Apodida investigated (Flammang & Conand, 2004) (Table 1). (*ii*) A ‘vesicular cell’ type has only been described in Apodida (Flammang & Conand, 2004) with unknown functions. (*iii*) Ciliated cells, also named ‘uniciliated cells’, ‘ciliated cells’ or ‘uniciliated sensory cells’ have been observed in all species investigated (Table 1). Two roles have been suggested for this cell type: they could mechanically disengage particles (Fankboner, 1978) or they could be sensory (Flammang & Conand, 2004). Bouland *et al*. (1982) associated the bud structure of *Holothuria* (*Panningothuria*) *forskali* Delle Chiaje with Laverack's (1974) description of chemosensory organs in marine invertebrates, where cilia are proposed to function as olfactory receptors, while microvilli are gustatory. Cilia are generally short and non‐motile (Dorsett & Hyde, 1969; Schulte & Riehl, 1976), like those depicted by Bouland *et al*. (1982). The apical elements of the tentacles are the first structures to contact substrates while the tentacles actively forage for food, strongly suggesting that the buds are likely to be chemosensory (Bouland *et al*., 1982). The cells of the epidermis of each bud are adjacent to the epineural nervous plate, which is connected to the hyponeural nerve plexus of the tentacles (Fig. 3E, F). Mesothelial muscles are present in each buccal tentacle (Fig. 3E; Bouland *et al*., 1982; McKenzie, 1987). (*iv*) ‘Mucous cells’, also called ‘mucocytes’ were observed in the tentacles of Dendrochirotida, Holothuriida and Apodida (Table 1). As similar cells are also observed outside the tentacles, their mucus‐secreting role is probably to ensure the presence of a physical barrier on the epidermis. (*v*) Type‐1 and (*vi*) type‐2 secretory cells are thought to play a direct role in the capture of food particles. Type‐1 secretory cells, also referred to as ‘granular cells’, ‘glandular vesicular cells’ or ‘papillate cells’ (Table 1) are characterised by numerous dense‐cored vesicles of 200–700 nm, with their diameter varying among species. These vesicles are found in many microvilli and are thought to be secreted into the cuticle. They may be homologues of the adhesive cells found in the podia of echinoderms. Type‐2 secretory cells, also named ‘type‐2 neurosecretory cells’ or ‘granular cells’, have been observed in Dendrochirotida and Apodida (Table 1). This cell type possesses numerous dense‐cored vesicles of 60–130 nm, again with their diameter varying among species. These vesicles are found in the cell apex and also are thought to be secreted into the cuticle. They are thought to be homologues of de‐adhesive cells found in the podia of echinoderms. Thus, the chemical capture of food particles could be performed by the secretion of type‐1 secretory cells and, when the tentacles are placed in the oral cavity, adhesion could be removed by the secretion of type‐2 secretory cells. This hypothesis is currently only theoretical and future functional morphology studies are needed, together with transcriptomic data to characterise the proteins expressed at the tentacle buds.

**Table 1:** Ultrastructural composition of the tentacle buds of holothuroids with the nomenclatural terms used in analyses by various authors. The first column describes the corresponding cells observed in podia of echinoderms where a duo glandular adhesive system (adhesion and de‐adhesion) has been identified

Authors	Fankboner (1978)	Bouland *et al*. (1982)	Smith (1983)	Cameron & Fankboner (1984)	McKenzie (1987)	Flammang & Conand (2004)
Analysed taxon	Dendrochirotida	Holothuriida	Dendrochirotida	Synallactida	Dendrochirotida	Apodida
Number of species studied	1	1	1	1	11	1
Cell with no particular development of intracellular component	**–**	**–**	**–**	**–**	Support cell	Support cell (T‐shaped cell) with 400**–**600 nm vesicles
Cell with large vesicle containing one spherule	**–**	**–**	**–**	**–**	**–**	Vesicular cell with numerous 4 μm vesicles
Cell with small apical cilia [presumed to be sensory cell, or cilia suggested to disengage food particles by Fankboner, 1978]	Uniciliated cell	Uniciliated cell	Ciliated cell	Uniciliated cell	Uniciliated cell	Uniciliated sensory cell
Cell filled with large clear vesicle (presumed to participate to the protection of the external surface	Mucous cell	Mucous cell	**–**	**–**	Mucous cell (two different types)	Mucocyte (goblet‐shaped cell) with 2 μm dense‐cored vesicles
Cell with large dense‐cored vesicles secreted into the cuticle (presumed to be similar to the adhesive cells identified in podia of echinoderms)	Papillary cell with 500 nm dense‐cored vesicles released into the cuticle	Glandular vesicular cell	Papillate cell with 300–600 nm dense‐cored vesicles released into the cuticle	Granular cell with 600–700 nm dense‐cored vesicles released into the cuticle	Type‐1 secretory cell with 200–400 nm dense‐cored vesicles released into the cuticle	Type‐1 secretory cell (spherical) with 250 nm dense‐cored vesicles
Cell with small dense‐cored vesicles (presumed to be similar to the de‐adhesive cells identified in podia of echinoderms)	**–**	**–**	Granular cell with 130 nm dense‐cored vesicles	**–**	Type‐2 presumed neurosecretory cell (due to low numbers) with 60–100 nm dense‐cored vesicles	Type‐2 secretory cell (spherical) with 90 nm dense‐cored vesicles

#### (b) Food particle ensnarement

Authors have long debated the mechanisms of food capture by which particles are trapped within bud interstices. Roberts (1979) suggested that expansion of the peltate tentacles caused inter‐bud spaces to open on the tentacles, which could then mechanically trap particles when the tentacles retract. Cameron & Fankboner (1984) reported mechanical ensnarement to be of relatively little importance for *Parastichopus californicus* [now *Apostichopus californicus* (Stimpson)] since there is no reverse process of tentacle expansion/relaxation while the tentacle is in the pharyngeal cavity. Food detection is likely to be related to the ciliated cells as described in Section II.2, as the tentacles spread onto the substratum or in the water column.

### (3) Digestive and assimilation processes in holothurians

#### (a) Digestive tract morphology

**Fig 4:**
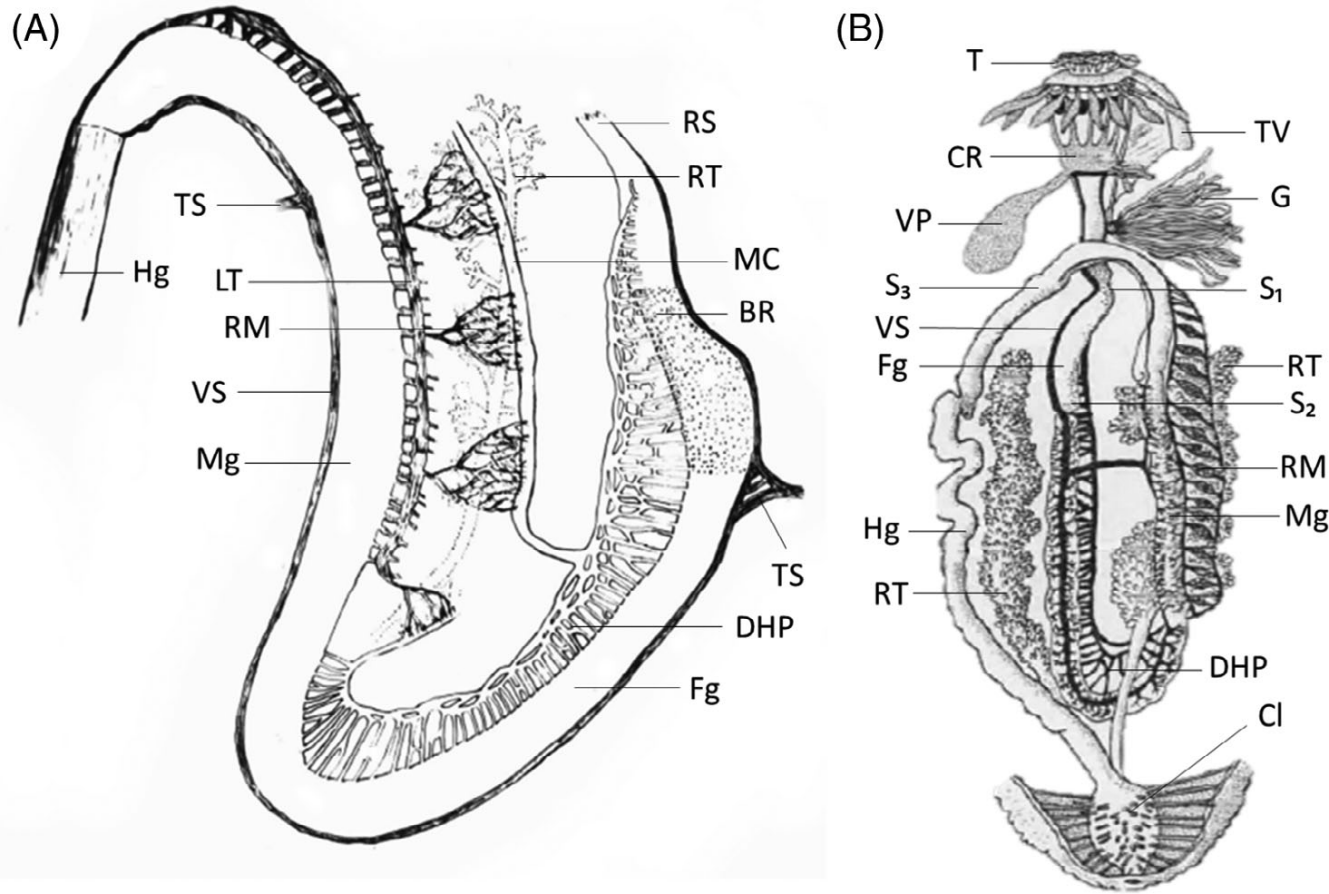
Digestive tract morphology of (A) *Holothuria atra* and (B) *H. tubulosa*. BR, brown region of the anterior foregut; Cl, cloaca; CR, calcareous ring; DHP, dorsal haemal plexus; Fg, foregut; G, gonads; Hg, hindgut; LT, longitudinal tube of rete mirabile; MC, main channel of dorsal haemal plexus; Mg, midgut; RM, rete mirabile; RS, region of severance; RT, respiratory tree; S_1_;, sphincter 1; S₂, sphincter 2; S₃, sphincter 3; T, tentacles; TS, transverse sinuses; TV, tentacle vesicles; VP, vesicle of Poli; VS, ventral sinus. Modified from Trefz (1958) and Massin (1978, inspired by Cuénot, 1948).

The nomenclature used for parts of the digestive tract varies among authors. Trefz (1958) distinguished between the mouth, pharynx, oesophagus, foregut, midgut, hindgut, cloaca and cloacal opening when studying the physiology of *Holothuria* (*Halodeima*) *atra* (Jaeger) (Fig. 4A). Massin (1978), in his study on holothurian nutrition, focused mainly on *Holothuria* (*Holothuria*) *tubulosa* Gmelin (Fig. 4B), differentiating the pharyngeal bulb surrounded by the calcareous ring, followed by the three portions of the digestive tract (foregut, midgut and hindgut), and finally the cloaca. Dividing the digestive tract between mouth and cloacal opening into three sections – foregut, midgut and hindgut – gives a broader view, and many studies describing holothurian feeding use this partitioning (e.g. Trefz, 1958; Ward‐Rainey, Rainey & Stackebrandt, 1996; Taddéi, 2006; Plotieau, 2012; Amaro *et al*., 2012).

The foregut includes the pharyngeal bulb, oesophagus, and the first descending loop of the intestine. The oesophagus is divided into two parts of equivalent lengths, both ending in a sphincter (sphincters 1 and 2) (Fig. 4B). The midgut begins immediately beyond the first intestinal loop where the proximal tubules of the *rete mirabile* are attached and consists of the ascending anterior loop of the intestine. A third sphincter separates the midgut from the hindgut. The hindgut, which represents approximately 70% of the total length of the gut, consists of the final posterior descending loop of the intestine and the cloaca from which respiratory trees emanate (Dolmatov & Ginanova, 2009). Only species from Pneumonophora have respiratory trees (Dendrochirotida, Holothuriida, Molpadida, Persiculida and Synallactida; Miller *et al*., 2017). The descending loop of the foregut is paralleled by the dorsal haemal plexus, which extends posteriorly to the anal end of the animal where it loops back. The dorsal haemal plexus is a complex of pulsating channels attached to the dorsal midline of the foregut. Longitudinally along the ventral side of this region runs the ventral sinus. The *rete mirabile* is a complex of tiny tubules, entangled within the respiratory tree, and attached to the ascending loop of the midgut. These tubules terminate into the main channel of the dorsal haemal plexus.

Although the foregut, midgut, and hindgut have three different functions (accumulation, digestion, and assimilation), most species of Synallactida, Persiculida, and Holothuriida are able to assimilate food from these three parts (Massin, 1978).

#### (b) Cell and enzymatic activities

The epithelia of the pharynx, oesophagus, and cloaca all contain T‐shaped cells that are involved in the uptake of dissolved organic matter. The gut epithelium is composed of enterocytes with a filamentous coat for intracellular digestion (Féral & Massin, 1982). A broad range of hydrolytic gut enzymes has evolved in association with the diet (Féral, 1989). Hydrolytic enzyme activity has been reported within the digestive tract, on cell membranes (Féral, 1989), and inside cells (Lawrence, 1982). Roberts *et al*. (2000) reviewed the enzymes found in the gut of deposit‐feeding holothurians (shallow‐water species in Holothuriida, Synallactida, Apodida and Dendrochirotida, and deep‐sea species of Holothuriida, Persiculida, Synallactida and Elasipodida), finding broad similarities in their hydrolytic enzymes. Gut enzymes include: (*i*) esterases that hydrolyse short‐chain fatty acids, with strong activity throughout the gut (Fish, 1967; Féral, 1989), (*ii*) lipases that hydrolyse long‐chain fatty acids, with lowest activity (Clifford *et al*., 1982; Féral, 1989; Manship, 1995), (*iii*) proteases, (*iv*) peptidases, which hydrolyse peptide bonds and may be important in the initial stages of protein digestion (Massin, 1984; Féral, 1989; Roberts *et al*., 2000), (*v*) saccharidases, and (*vi*) phosphatases, present at high concentrations in the gut tissue (Féral, 1989; Boetius & Felbeck, 1995).

Trefz (1958) observed phagocytic activity of abundant cells in the holothurian gut, such as round phagocytic amoebocyte cells that ingest and digest bacteria (*Bacillus subtilis*), but not indigestible particles.

#### (c) Obtaining nutrients through cloacal water retention

The major functions of cloacal ventilation are commonly described as respiration, excretion, and salt balance. Holothurians with respiratory trees (absent in Apodida and Elasipodida) have the ability to pump large volumes of water into and out of the cloacal opening. A similar pumping mechanism is reported for echinoderms such as holothuroids (Newell & Courtney, 1965; Brown & Shick, 1979) and edrioasteroids (Bell, 1977), annelids (Wolcott, 1981), and crustaceans (Fox, 1952).

Innovative work, using radiography and stable isotope enrichment, demonstrated that the epithelium of the respiratory tree can assimilate dissolved organic matter such as monosaccharides and amino acids from the water column during cloacal water retention (Fontaine & Chia, 1968; Brothers, Lee & Nestler, 2015). The respiratory tree of *Cucumaria lactea* [now *Ocnus lacteus* (Forbes & Goodsir)] can assimilate dissolved ^3^H‐labelled glycine and ^3^H‐labelled glucose (Fontaine & Chia, 1968). The respiratory tree of *A. californicus* assimilated ^15^N‐labelled amino acids and peptides (Brothers *et al*., 2015) and ^14^C‐labelled unicellular algae, suggesting the transfer of nutrients from the respiratory tree into the haemal system (Jaeckle & Strathmann, 2013). Thus, is possible that holothurians can obtain nutrients through their respiratory trees as a result of cloacal pumping, but additional studies are needed to clarify its importance to these animals.

## III. HOLOTHURIAN FEEDING BEHAVIOUR

As for all organisms, the movements and behaviour of holothurians will be driven by physiological requirements such as feeding, reproduction, or avoidance of stress factors (Mercier, Battaglene & Hamel, 1999; Pitt & Duy, 2004; Meng *et al*., 2011; Hamel *et al*., 2019). A possible role of pheromones in intra‐ or inter‐specific control of reproduction was recently hypothesised for holothurians (Marquet *et al*., 2018).

### (1) Deposit‐ or suspension‐feeding

The first larval stage in holothurians, the auricularia, feeds on phytoplankton (planktotrophic) by means of cilia located on the epidermis. The second larval stage, the doliolaria, is non‐feeding, and the final stage, the pentactula, develops the tentacles that are present in juveniles and adults. The first and second stages can be absent in holothurians with direct development (McEdward & Miner, 2001; Raff & Byrne, 2006). In general, juveniles and adults are particle‐feeders, which usually exhibit either suspension‐ (in Dendrochirotida) or deposit‐feeding (most other taxa) behaviour (Roberts *et al*., 2000; Pawson, 1970; Bakus, 1973). Suspension‐feeding holothurians tend to live in high‐energy environments to allow them to intercept food particles (Taghon & Jumars, 1984). Deposit‐feeding behaviour can be subdivided into three groups related to feeding depth (Roberts *et al*., 2000). (*i*) Epibenthic deposit‐feeders feed at the interface between the water column and sediment, or on the surface of seagrasses (Cuvillier, 2016), the sand scattered over the tegument of other specimens (e.g. *H. atra*; J.P., personal observations), boulders and corals (Roberts *et al*., 2000; J.P., personal observations), or sponges (Hammond & Wilkinson, 1985). (*ii*) Subsurface deposit‐feeders feed below the sediment surface. (*iii*) Funnel deposit‐feeders create funnel‐shaped depressions in sediments and feed on particles trapped in these funnels (Jumars, 1993).

Feeding mode may vary at the individual or population scale. According to Cadée (1984), many benthic individuals are opportunistic in their feeding mode. Some individuals of species in Dendrochirotida (Roberts *et al*., 2000), Synallactida, Persiculida, and Holothuriida (Da Silva, Cameron & Fankboner, 1986) can shift between deposit‐feeding and suspension‐feeding. This may be possible if the individual is positioned in a negative geotropic orientation (Da Silva *et al*., 1986). *Leptopentacta elongata* (Düben & Koren) and other shallow‐water species (Fankboner, 1981; Levin, 1989) are known to shift opportunistically from suspension‐ to deposit‐feeding when the concentration of suspended particulate organic matter is low. This opportunistic behaviour is driven by environmental factors such as food pulses in shallow‐water (tidal or seasonal fluctuations) (Cushing, 1959) and deep‐sea ecosystems (Jumars, Self & Nowell, 1982; Billett *et al*., 1983).

At the population scale, the feeding modes of some holothurian species can vary depending on their geographic location. *Holothuria (Thymiosycia) arenicola* Semper is an epibenthic feeder, feeding on surface layers of sand under coral debris in the Indo‐West Pacific region and Cuba (Levin, 1989), whereas in the Bahamas, it feeds on particles in sediments (Mosher, 1980) as a subsurface deposit‐feeder. The underlying reasons for these intraspecific differences at a population scale, and their drivers, are poorly studied.

### (2) Daily burrowing cycle

A study on the burrowing behaviour of shallow‐water holothurians in Palao Island classified holothurians into two groups according to their feeding habits (Yamanouchi, 1939): (*i*) species that do not burrow into sediments or other substrates and feed continuously, such as *Holothuria* (*Halodeima*) *edulis* Lesson, *H*. (*Semperothuria*) *flavomaculata* Semper or *H. atra* (Yamanouchi, 1939; Trefz, 1958; Uthicke, 1994) and (*ii*) species that show a daily cycle of burrowing and feeding (Yamanouchi, 1939, 1956; Mercier *et al*., 1999; Lavitra *et al*., 2009). The factors regulating burrowing and feeding cycles appear to be complex (Yamanouchi, 1939, 1956), with differences among species increasing the complexity of cues that drive such behaviour and impeding generalisation.

According to Yamanouchi (1956), most adults prefer to feed during the day, particularly in the early afternoon when the water temperature is highest (Mercier *et al*., 1999) and productivity of the marine ecosystems is maximal (Heil *et al*., 2004). Some species, such as *Stichopus chloronotus* Brandt, move under corals from 00:00 to 10:00 and then return to sediments to feed during the day (Yamanouchi, 1956). Similarly, *Stichopus variegatus* (now *Stichopus herrmanni* Semper) hides under seagrasses between 20:00 and 10:00 (Yamanouchi, 1956). Coulon & Jangoux (1993) argued that it may be more energetically advantageous for juveniles of *H. tubulosa* to stop feeding during the coldest hours of the day. Individuals exposed to abnormal water temperatures exhibit unusual behaviour and feeding activity (Kato & Hirata, 1990). The temperate species *A. japonicus* aestivates during the warmest months when water temperatures are between 20 and 24.5°C (Choe, 1963), especially large and mature individuals (Liu *et al*., 1996). Aestivating individuals hide under structures and enter a state of dormancy (Liu *et al*., 1996; Yang *et al*., 2005) in which feeding activity ceases (Yuan *et al*., 2007) until water temperature becomes more favourable. During aestivation, *A. japonicus* individuals undergo a series of physiological and morphological changes (Wang *et al*., 2008), losing 30–50% of their body mass (Liu *et al*., 1996). The digestive tract degenerates to half its pre‐aestivation size (Li *et al*., 1996; Liu *et al*., 1996). Physiological responses associated with digestion are reduced during aestivation, with lower activity of digestive enzymes in the principal portions of the gut (Cui, Dong & Lu, 2000). Similarly, under high temperatures (austral summer), *Holothuria* (*Metriatyla*) *scabra* Jaeger, changes its burrowing behaviour, remaining on the sediment surface and feeding frequently, whereas at low water temperatures (austral winter) it remains beneath the sediment surface for most of the day (Mercier, Battaglene & Hamel, 2000). Thus, seasonal patterns will play a major role in the regulation of the daily burrowing cycle of this species.

The effects of salinity on the foraging activities of holothurians are unclear. Mercier *et al*. (1999, 2000) found that *H. scabra* cease feeding when water salinity is lowest, and burrow into the substrate. James & James (1994) reported conflicting results, with juveniles emerging during low tide. Skewes *et al*. (2006) reported that only a third of *H. scabra* adults emerge during high tide.

Some studies show conflicting results concerning daily burrowing cycles of juveniles and adults. On one hand, daily burrowing cycles can be similar for juveniles and adults of the same species. In Palao Island, *H. scabra* adults were observed burrowing in the sediment between 03:30 and 15:00 and feeding between 15:00 and 03:30 (Yamanouchi, 1956). In the Philippines, *H. scabra* juveniles burrow in sediments between 03:00 and 09:00 and remain hidden until they emerge to feed between 15:00 to 03:00 (Altamirano, Recente & Rodriguez Jr., 2017). Despite their different life stages and different geographical regions, both species therefore feed during the same time periods. On the other hand, holothurian size has been reported to affect burrowing and daily cycles. Mercier *et al*. (1999) found the burrowing cycle of *H. scabra* to be driven by light for small juveniles (10–40 mm), which burrow at sunrise and emerge close to sunset, whereas temperature was more influential for intermediate‐sized juveniles (40–140 mm), which emerge earlier in the afternoon. Like many echinoderms, the nocturnal feeding and movement habits of smaller holothurians might be an adaptation to avoid predation (Nelson & Vance, 1979; Hammond, 1982).

### (3) Selectivity by deposit‐feeding holothurians

Feeding selectivity is observed in many marine species of different taxa, such as Polychaeta (Petch, 1986; Shimeta, 1996), Gastropoda (Whitlatch & Obrebski, 1980), or Bivalvia (Hylleberg & Gallucci, 1975). Some other echinoderms show selectivity in feeding, including species in Asteroidea (Mellin *et al*., 2017) and Echinoidea (Larson, Vadas & Keser, 1980; Boon & Duineveld, 2012).

To determine particle selectivity in deposit‐feeding holothuroids, most studies compare the biochemical composition and concentration of compounds present within sediments around and beneath foraging individuals and in their foregut (Moriarty, 1982; Hammond, 1983; Amaro *et al*., 2010). Examining particle selectivity is subject to the difficulty of investigating sediment ingestion at the appropriate scale (Lopez & Levinton, 1987). Particle selection is generally based on size, surface texture, specific gravity, and the presence of an organic coating (Taghon, 1989), but it can also be explained by mechanical processes (Jumars *et al*., 1982). Discussion of particle selectivity by deposit‐feeding holothurians usually refers to two different modes: the selection of particles with a specific grain size or selection of those with higher organic content. We found 29 studies describing both aspects of particle selectivity for shallow‐water (23 studies) and deep‐sea (six studies) holothurian species (Table 2). A total of 50 species have been studied, with only one study focused on suspension‐feeding species (Hamel & Mercier, 1998).

**Table 2:** Synthesis of studies on selective feeding strategies in shallow‐water and deep‐sea holothurians

Taxon	Size particle selectivity	Organic matter selectivity	Location	Authors
*Coastal species*				
**> Apodida**				
*Euapta lappa*	No	Not studied	Discovery Bay, Jamaica	Hammond (1982)
*Leptosynapta tenuis*	No	Not studied	North Carolina, USA	Powell (1977)
**> Holothuriida**				
*Actinopyga agassizi*	No	Not studied	Discovery Bay, Jamaica	Hammond (1982)
*Bohadschia bivittata*	Yes, 400 μm	Not studied	Pari Island, Indonesia	Roberts (1979)
*Bohadschia vitiensis*	Yes, gravel and coarse particles (spawning period: fine particles)	Yes	Hurghada, Egypt	Dar & Ahmad (2006)
*Holothuria arenicola*	No	Not studied	Discovery Bay, Jamaica	Hammond (1982)
*Holothuria atra*	Not studied	No	Great Palm Island, Australia	Uthicke & Karez (1999)
	Yes, coarser particles	Yes	Red Sea coast, Egypt	Dar (2004)
	Yes	Not studied	Waikiki branch, Hawaï	Trefz (1958)
	Yes, gravel and coarse particles (in spawning period fine particles)	Yes	Hurghada, Red Sea, Egypt	Dar & Ahmad (2006)
	Not studied	Yes	Great Barrier Reef, Australia	Moriarty (1982)
	Yes, coarser particles	Yes	El Qasr reef, Saudi Arabia	Behairy, Beltagi & Rao (1985)
	Yes, 350 μm	Not studied	Pari Island, Indonesia	Roberts (1979)
*Holothuria cinerascens*	Yes, <63–500 μm	Not studied	Beacon Island, Australia	Roberts & Bryce (1982)
*Holothuria edulis*	Not studied	No	Great Palm Island, Australia	Uthicke & Karez (1999)
	Yes, 63–125 μm	Not studied	Beacon Island, Australia	Roberts & Bryce (1982)
*Holothuria forskali*	Yes, 60–200 μm	Yes	Algiers and Bou‐Ismail Bay, Algeria	Mezali & Soualili (2013)
	Not studied	Yes	Toulon, France	Massin & Jangoux (1976)
*Holothuria grisea*	No	Not studied	Discovery Bay, Jamaica	Hammond (1982)
*Holothuria hartmeyeri*	Yes, 2000–3500 μm	Not studied	Beacon Island, Australia	Roberts & Bryce (1982)
*Holothuria hawaiiensis*	Yes, gravel and coarse particles (in spawning period fine particles)	Yes	Hurghada, Egypt	Dar & Ahmad (2006)
*Holothuria impatiens*	Yes, <63–500 μm	Not studied	Beacon Island, Australia	Roberts & Bryce (1982)
*Holothuria leucospilota*	Yes, coarse particles	Yes	Red Sea coast, Egypt	Dar (2004)
*Holothuria marmorata*	Yes, coarse particles	Yes	Red Sea coast, Egypt	Dar (2004)
*Holothuria mexicana*	No	Not studied	Discovery Bay, Jamaica	Hammond (1982)
*Holothuria nobilis*	Not studied	No	Great Palm Island, Australia	Uthicke & Karez (1999)
	Yes, 2000–3500 μm	Not studied	Beacon Island, Australia	Roberts & Bryce (1982)
*Holothuria cf. pervicax*	Yes, 2000–3500 μm	Not studied	Beacon Island, Australia	Roberts & Bryce (1982)
*Holothuria poli*	Yes, 200–600 μm	Yes	Algiers and Bou‐Ismail Bay, Algeria	Mezali & Soualili (2013)
	No	Yes	Toulon, France	Massin & Jangoux (1976)
*Holothuria sanctori*	Yes, 60–200 μm	Yes	Algiers and Bou‐Ismail Bay, Algeria	Mezali & Soualili (2013)
	Not studied	Yes	Canary Islands, Spain	Navarro *et al*. (2013)
*Holothuria scabra*	Yes, 125–250 μm	Not studied	Palk Bay, India	Baskar (1994)
*Holothuria stellati*	Yes, 60–200 μm	No	Algiers and Bou‐Ismail Bay, Algeria	Mezali & Soualili (2013)
*Holothuria tubulosa*	Yes, 200–600 μm	Yes	Algiers and Bou‐Ismail Bay, Algeria	Mezali & Soualili (2013)
	Not studied	Yes	Gulf of Naples, Italy	Amon & Herndl (1991)
	No	Yes	Toulon, France	Massin & Jangoux (1976)
**> Molpadida**				
*Molpadia oolitica*	Yes, smallest particles	Not studied	Cape Cod Bay, USA	Rhoads & Young (1971)
**> Synallactida**				
*Australostichopus mollis*	No	Yes	Mahurangi Harbour, New Zealand	Slater, Jeffs & Sewell (2011)
*Isostichopus badionotus*	No	Yes	Bermuda	Sloan & von Bodungen (1980)
	No	Not studied	Discovery Bay, Jamaica	Hammond (1982)
*Parastichopus californicus*	Not studied	Yes	British Columbia, Canada	Paltzat *et al*. (2008)
*Parastichopus parvimensis*	No	Yes	Santa Catalina Island, USA	Yingst (1976)
*Stichopus chloronotus*	Not studied	Yes	Great Palm Island, Australia	Uthicke & Karez (1999)
	Not studied	Yes	Lizard Island, Australia	Uthicke (1999)
	Not studied	Yes	Great Barrier Reef, Australia	Moriarty (1982)
*Stichopus japonicus*	Not studied	Yes	Aquarium experiment, Japan	Michio *et al*. (2003)
*Stichopus tremulus*	Yes, coarse particles	Yes	Raunefjorden, Norway	Hauksson (1979)
*Stichopus variegatus*	Not studied	Yes	Great Palm Island, Australia	Uthicke & Karez (1999)
*Deep‐sea species*				
**> Apodida**				
*Chiridota* sp.	Not studied	No	Santa Catalina Basin and Hawaiian slope	Miller *et al*. (2000)
**> Elasipodida**				
*Amperima rosea*	Not studied	Yes	Porcupine Abyssal Plain, NE Atlantic	Wigham *et al*. (2003)
	Not studied	Yes	Porcupine Abyssal Plain, NE Atlantic	Ginger *et al*. (2001)
*Benthogone rosea*	Yes, 7–14 μm	Yes	Golfe de Gascogne, France	Khripounoff & Sibuet (1980)
*Ellipinion molle*	Not studied	Yes	Porcupine Abyssal Plain, NE Atlantic	Ginger *et al*. (2001)
*Pannychia moseleyi*	Not studied	Yes	Santa Catalina Basin and Hawaiian slope	Miller *et al*. (2000)
*Peniagone vignoni*	Not studied	No	West Antarctic Peninsula	Wigham *et al*. (2008)
*Protelpidia murrayi*	Not studied	No	West Antarctic Peninsula	Wigham *et al*. (2008)
*Psychropotes longicauda*	Yes, 6.2–44 μm	Yes	Golfe de Gascogne, France	Khripounoff & Sibuet (1980)
	Not studied	Yes	Porcupine Abyssal Plain, NE Atlantic	Wigham *et al*. (2003)
*Scotoplanes globosa*	Not studied	Yes	Santa Catalina Basin and Hawaiian slope	Miller *et al*. (2000)
**> Holothuriida**				
*Mesothuria carnosa*	Not studied	Yes	Santa Catalina Basin and Hawaiian slope	Miller *et al*. (2000)
**> Molpadida**				
*Molpadia blakei*	Not studied	Yes	Porcupine Abyssal Plain, NE Atlantic	Wigham *et al*. (2003)
	No	Not studied	Golfe de Gascogne, France	Khripounoff & Sibuet (1980)
*Molpadia musculus*	Not studied	No	West Antarctic Peninsula	Wigham *et al*. (2008)
**> Persiculida**				
*Paroriza pallens*	Yes, 8–54 μm	Yes	Golfe de Gascogne, France	Khripounoff & Sibuet (1980)
*Pseudostichopus villosus*	Not studied	Yes	Porcupine Abyssal Plain, NE Atlantic	Wigham *et al*. (2003)
*Pseudostichopus sp*.	Not studied	Yes	Porcupine Abyssal Plain, NE Atlantic	Wigham *et al*. (2003)
	Not studied	No	West Antarctic Peninsula	Wigham *et al*. (2008)
**> Synallactida**				
*Paelopatides retifer*	Not studied	No	Santa Catalina Basin and Hawaiian slope	Miller *et al*. (2000)
*Oneirophanta mutabilis*	Not studied	Yes	Porcupine Abyssal Plain, NE Atlantic	Witbaard *et al*. (2001)
	Not studied	Yes	Porcupine Abyssal Plain, NE Atlantic	Wigham *et al*. (2003)

#### (a) Size selection of particles

Whether deposit‐feeding holothurians exhibit a preference for substrates with a specific grain size is still debated (Mercier *et al*., 2000), with conflicting results available (Table 2) even within a single species. For example, Mezali & Soualili (2013) stated that *H. tubulosa* and *Holothuria* (*Roweothuria*) *poli* Dell Chiaje select a grain size ranging between 200 and 600 μm, whereas Massin & Jangoux (1976) found no size preference for these same species. Some authors conclude that holothurians are unselective regarding the size of ingested particles because ingested sediment particles tend to be very similar in size to those of the bottom sediment (Yingst, 1976; Sloan & von Bodungen, 1980; Hammond, 1982). Trefz (1958) noted that diverse holothurian species forage and subsist on different substrates, suggesting that they may focus their feeding on specific substrates. Of the 29 shallow‐water species evaluated to date, 20 show grain size selectivity (Table 2); among the four deep‐sea species studied, only *Molpadia blakei* (Théel) is unselective (Table 2; Khripounoff & Sibuet, 1980).

Particle‐size selection seems to be species dependent: preferred grain size is 200–600 μm for *H. tubulosa* (Mezali & Soualili, 2013), 125–250 μm for *H. scabra* (Baskar, 1994), and 2000–3500 μm for *H. nobilis* (Roberts & Bryce, 1982). Deep‐sea species seem to prefer finer particles than shallow‐water species, with a median of 7–14 μm for *Benthogone rosea* Koehler and 8–54 μm for *Paroriza pallens* (Koehler) (Khripounoff & Sibuet, 1980). Particle‐size selection may change within a species depending on the season: *Holothuria* (*Stauropora*) *hawaiiensis* Fisher and *Bohadschia vitiensis* (Semper) prefer finer particles during the spawning period and gravel and coarse particles during the rest of the year (Dar & Ahmad, 2006).

More information is required to assess the benefits of feeding on small‐grain sediments for holothurians and the reasons why only some species are selective. Although the mechanisms by which a preferred substrate is selected are not understood, Roberts (1979) proposed that this could be associated with the morphology of the oral tentacles.

#### (b) Selection of organically rich particles

A wide range of holothurian species from tropical, shallow‐water regions (Moriarty, 1982; Hammond, 1983; Uthicke, 1999; Uthicke & Karez, 1999), temperate seas (Hauksson, 1979; Amon & Herndl, 1991), and deep seas (Miller *et al*., 2000; Wigham *et al*., 2003, 2008) are known to select organically rich particles from the sediment (Table 2). These species seem to be attracted by sediments with high organic content (Yingst, 1982) and can differentiate and capture the preferred particles (Massin & Jangoux, 1976; Moriarty, 1982). Out of the 37 species studied, 29 prefer particles enriched in organic matter (Table 2). Both shallow‐water and deep‐sea species seem to be highly selective, with 17 out of 20 (85%) and 12 out of 17 species (70%), respectively, showing selectivity for organic matter (Table 2).

Generally, the organic matter content of the ingested sediment is much higher than in the sediment surrounding the animal (Hauksson, 1979). Khripounoff & Sibuet (1980), studying the selective feeding of four abyssal species (*Psychropotes longicauda* Théel, *P. pallens*, *B. rosea* and *M. blakei*), found that with concentrations of organic carbon and nitrogen in the foregut were four and six times greater, respectively, than concentrations in the local sediment.

Moriarty (1982) suggested that particle size selection in holothurian deposit‐feeders might be explained by a non‐uniform distribution of organic matter in sediments. Uthicke (1999) supported this hypothesis, arguing that a higher organic content is usually associated with smaller sediment grains with low specific gravity, which a richer host microflora (bacteria, microalgae, etc.), due to their higher surface area to volume ratio (Johnstone, Koop & Larkum, 1990). All studies, with one exception [for *Holothuria* (*Holothuria*) *stellati* Delle Chiaje (Mezali & Soualili, 2013)], that showed a preference for a particular grain size also found a preference for organically rich particles where this was investigated (Table 2). A relationship between the organic load and particle size could therefore explain the confusion between these two selective strategies of deposit‐feeders.

#### (c) Patch selectivity

Patch selectivity describes the preference of a mobile organism to feed on patches in a heterogeneous environment (Uthicke & Karez, 1999). A patchy distribution of shallow‐water holothurians, thought to be related to their feeding habits (Uthicke & Karez, 1999), has been described in ecosystems including Milne Bay Province and Torres Strait islands (Skewes *et al*., 2002, 2006), the Gulf of Mannar in India (Asha *et al*., 2015), New Caledonia (Purcell *et al*., 2009), and most parts of the western Indian Ocean (Conand, 2008). For holothurians, the energetic cost of processing poor sediment is higher than the cost of moving to a more suitable feeding substrate (Mercier *et al*., 1999). Mercier *et al*. (1999) found that the locomotive speed of *H. scabra* juveniles was higher on substrates with low organic matter content (71–331 cm day^−1^) than on substrates of better quality (150–215 cm day^−1^), reflecting an active search for organically rich sediments. Similar results were reported for *H. atra* and *S. variegatus* (now *S. herrmanni*), which move 0–52 m per day and cover greater distances on poorer feeding areas (Yamanouchi, 1939). Other studies on *Cucumaria frondosa* (Gunnerus) (Hamel & Mercier, 1998), *P. californicus* (now *A. californicus*) (Cameron & Fankboner, 1984), *Parastichopus chitonoides* (Young & Chia, 1982), *Actinopyga echinites* (Jaeger) (Wiedemeyer, 1994), and *H. scabra* (James & James, 1994; Altamirano *et al*., 2017) generally describe the quality of the substrate where holothurians were observed but do not detail optimal substrates in terms of nutritional or ecological features (e.g. optimal grain size, organic matter content, origin of sediments, presence of conspecifics or optimal light regime).

A patchy distribution also has been observed in deep‐sea ecosystems for several species (Billett, Llewellyn & Watson, 1988; Ruhl & Smith, 2004) that feed preferentially on nutritionally rich food patches (Hauksson, 1979; Hudson *et al*., 2005; Jamieson *et al*., 2011). Particulate organic carbon deposited on the seabed is considered an important factor controlling the local abundance and composition of macrofauna and megafauna (Sibuet, 1985; De Leo *et al*., 2010). Significant correlations have been found between food availability and megafaunal abundance, particularly for holothurians (Billett *et al*., 2001; Ruhl & Smith, 2004). Preferences for selective feeding amongst holothurian species may therefore function in niche partitioning (Roberts, 1979; Sloan & von Bodungen, 1980; Massin & Doumen, 1986), with reduced intra‐ and inter‐specific competition as a result (Jamieson *et al*., 2011).

## IV. THE SEDIMENT INGESTED BY HOLOTHURIANS: A COMPLEX FOOD SOURCE

Despite much published work on holothurians, very few studies have focused on the quality of their food. Table 3 provides a list of known food sources for holothurians, of both organic and inorganic origin. We divide these food sources into two categories: living and non‐living fractions. Living fractions include organisms associated with sediments such as bacteria, photosynthetic organisms or meiofauna. Non‐living fractions include organically derived detrital matter such as phytodetritus, dead and decaying animals, and faecal pellets and inorganic compounds such as coral scraps, shell remains, coralline algae, foraminiferal tests, and silicates. Sadly, holothurians have not escaped the increasing pollution of the ocean, with ingestion of plastic particles or microplastics recorded (Graham & Thompson, 2009; Renzi *et al*., 2018).

**Table 3:** Food sources recorded as ingested by coastal and deep‐sea holothurians

Food sources ingested	Indicator studied	Authors	Order	Species studied	Habitat	Number of individuals studied
Living fractions						
Bacteria	Abundance	Deming & Colwell (1982)	Synallactida	*Deima* sp.	Deep sea	2
			Persiculida	*Pseudostichopus* sp.	Deep sea	3
		Roberts *et al*. (2001)	Persiculida	*Molpadiodemas villosus*	Deep sea	Up to 25
			Synallactida	*Oneirophanta mutabilis*	Deep sea	Up to 25
			Elasipodida	*Psychropotes longicauda*	Deep sea	Up to 25
		Taddéi (2006)	Holothuriida	*Holothuria atra*	Coastal	18
				*Holothuria leucospilota*	Coastal	7
	Abundance and diversity	Amaro *et al*. (2012)	Molpadida	*Molpadia musculus*	Deep sea	20
		Plotieau *et al*. (2013)	Holothuriida	*Holothuria scabra*	Coastal	4 for abundance
						30 for diversity
		Ward‐Rainey *et al*. (1996)	Holothuriida	*H. atra*	Coastal	2
	Diversity	Amaro *et al*. (2009)	Molpadida	*M. musculus*	Deep sea	15
		Gao *et al*. (2017)	Synallactida	*Apostichopus japonicus*	Coastal	240
		Sha *et al*. (2016)	Synallactida	*A. japonicus*	Coastal	30
		Zhang *et al*. (2012)	Holothuriida	*H. leucospilota*	Coastal	2
Photosynthetic organisms	Abundance	Taddéi (2006)	Holothuriida	*H. atra*	Coastal	18
				*H. leucospilota*	Coastal	6
	Abudance and diversity	Belbachir & Mezali (2018)	Holothuriida	*Holothuria forskali*	Coastal	10
				*Holothuria poli*	Coastal	10
				*Holothuria sanctori*	Coastal	10
				*Holothuria tubulosa*	Coastal	10
		Hamel & Mercier (1998)	Dendrochirotida	*Cucumaria frondosa*	Coastal	20
		Hamel, Himmelman & Dufresne (1993)	Dendrochirotida	*Psolus fabricii*	Coastal	30
		Kang *et al*. (2008)	Holothuriida	*H. atra*	Coastal	NA
		Khripounoff & Sibuet (1980)	Elasipodida	*Benthogone rosea*	Deep sea	NA
				*P. longicauda*	Deep sea	NA
			Molpadida	*Molpadia blakei*	Deep sea	NA
			Persiculida	*Paroriza pallens*	Deep sea	NA
		Sonnenholzner (2003)	Holothuriida	*Holothuria theeli*	Coastal	200
		Tyler *et al*. (1992)	Persiculida	*P. pallens*	Deep sea	52
		Uthicke (1999)	Holothuriida	*H. atra*	Coastal	6
			Synallactida	*Stichopus chloronotus*	Coastal	6
Meiofauna	Abundance and diversity	Belbachir & Mezali (2018)	Holothuriida	*H. forskali*	Coastal	10
				*H. poli*	Coastal	10
				*H. sanctori*	Coastal	10
				*H. tubulosa*	Coastal	10
		Hamel & Mercier (1998)	Dendrochirotida	*C. frondosa*	Coastal	20
		Kang *et al*. (2008)	Holothuriida	*H. atra*	Coastal	NA
		Khripounoff & Sibuet (1980)	Elasipodida	*B. rosea*	Deep sea	NA
				*P. longicauda*	Deep sea	NA
			Molpadida	*M. blakei*	Deep sea	NA
			Persiculida	*P. pallens*	Deep sea	NA
		Sonnenholzner (2003)	Holothuriida	*H. theeli*	Coastal	200
		Tyler *et al*. (1992)	Persiculida	*P. pallens*	Deep sea	52
		Uthicke (1999)	Holothuriida	*H. atra*	Coastal	6
			Synallactida	*S. chloronotus*	Coastal	6
Non‐living fractions						
Detrital matter	Abundance	Suchanek *et al*. (1985)	Holothuriida	*Mesothuria verrilli*	Deep sea	5
			Elasipodida	*Benthodytes lingua*	Deep sea	2
				*Psychropotes semperiana*	Deep sea	1
	Abudance and diversity	Belbachir & Mezali (2018)	Holothuriida	*H. forskali*	Coastal	10
				*H. poli*	Coastal	10
				*H. sanctori*	Coastal	10
				*H. tubulosa*	Coastal	10
		Costa, Mazzola & Vizzini (2014)	Holothuriida	*H. tubulosa*	Coastal	3
		Khripounoff & Sibuet (1980)	Elasipodida	*B. rosea*	Deep sea	NA
				*P. longicauda*	Deep sea	NA
			Molpadida	*M. blakei*	Deep sea	NA
			Persiculida	*P. pallens*	Deep sea	NA
	Diversity	Hammond & Wilkinson (1985)	Apodida	*Synaptula lamperti*	Coastal	40
Minerals	Abundance and diversity	Belbachir & Mezali (2018)	Holothuriida	*H. forskali*	Coastal	10
				*H. poli*	Coastal	10
				*H. sanctori*	Coastal	10
				*H. tubulosa*	Coastal	10
		Khripounoff & Sibuet (1980)	Elasipodida	*B. rosea*	Deep sea	NA
				*P. longicauda*	Deep sea	NA
			Molpadida	*M. blakei*	Deep sea	NA
			Persiculida	*P. pallens*	Deep sea	NA
		Tyler *et al*. (1992)	Persiculida	*P. pallens*	Deep sea	52
	Diversity	Plotieau (2012)	Holothuriida	*H. scabra*	Coastal	4
Plastic particles	Abundance and diversity	Graham & Thompson (2009)	Dendrochirotida	*C. frondosa*	Coastal	42
				*Thyonella gemmata*	Coastal	30
			Holothuriida	*Holothuria grisea*	Coastal	46
				*Holothuria floridana*	Coastal	53
		Iwalaye, Moodley & Robertson‐Andersson (2020)	Holothuriida	*Holothuria cinerascens*	Coastal	20
		Mohsen *et al*. (2019)	Synallactida	*A. japonicus*	Coastal	65
		Renzi *et al*. (2018)	Holothuriida	*H. tubulosa*	Coastal	30

### (1) The living fractions

#### (a) Bacteria

The total biomass of bacteria is relatively high in shallow‐water and deep‐sea sediments (Zobell & Morita, 1959; Danovaro, Fabiano & Della Croce, 1993; Danovaro *et al*., 1998; Rex *et al*., 2006). Most benthic bacteria are not suspended in interstitial water but are attached to mineral or organic sediment particles (Dale, 1974) in aggregates and colonies (Taddéi, 2006). Thus, bacteria could represent a major food source for deposit‐feeders (Sorokin, 1972; Massin, 1982) such as holothurians. We found only 10 articles that studied bacteria in the diet of deposit‐feeding holothurians, of which four focused on the diversity of ingested bacteria, three on their abundance, and three on both. To our knowledge, the abundance and diversity of bacteria in the gut of suspension‐feeding holothurians has not been assessed.

Most studies found a similar distribution pattern of bacteria in the digestive tract for coastal (Taddéi, 2006; Plotieau *et al*., 2013) and deep‐sea holothurian species (Deming & Colwell, 1982; Roberts *et al*., 2001; Amaro *et al*., 2012): an increase in bacterial abundance between the sediment and the foregut contents, then a decrease between the foregut and the hindgut. For example, Taddéi (2006), studying two coastal species *H. atra* and *Holothuria* (*Mertensiothuria*) *leucospilota* (Brandt), found an abundance of 1.50 × 10^7^ bacteria g^−1^ in coral reef shallow‐water sediments of La Réunion. This increased to 3.66 × 10^7^ bacteria g^−1^ in the foregut of *H. atra* and 4.46 × 10^7^ bacteria g^−1^ in the foregut of *Holothuria leucospilota*, which decreased significantly to 1.10 × 10^7^ and 1.01 × 10^7^ bacteria g^−1^, respectively, in the hindgut. Bacterial abundance was similar in the faeces (1.91 × 10^7^ and 0.87 × 10^7^ bacteria g^−1^). Taddéi (2006) reported that about 53% of bacteria are digested by *H. atra*. Plotieau *et al*. (2013) reported a higher abundance of 11 × 10^9^ bacteria g^−1^ in the foregut of the coastal species *H. scabra*, which again decreased in the midgut (4 × 10^9^ bacteria g^−1^) and remained stable in the faeces. They estimated that *H. scabra* digested up to 59% of the ingested bacteria. Amaro *et al*. (2012) reported a value of 80% for *Molpadia musculus* Risso.

Ward‐Rainey *et al*. (1996) found the opposite pattern for the two specimens of *H. atra* that they studied. The abundance of bacteria decreased from 3 × 10^4^ and 3 × 10^6^ colony forming units (cfu) to 3.4 × 10^3^ and 6.2 × 10^4^ cfu, respectively, between the sediment and the foregut, and then increased to 3 × 10^4^ and 1.8 × 10^6^ cfu in the hindgut. However, the low number of replicates means that these results should be considered with caution.

**Fig 5:**
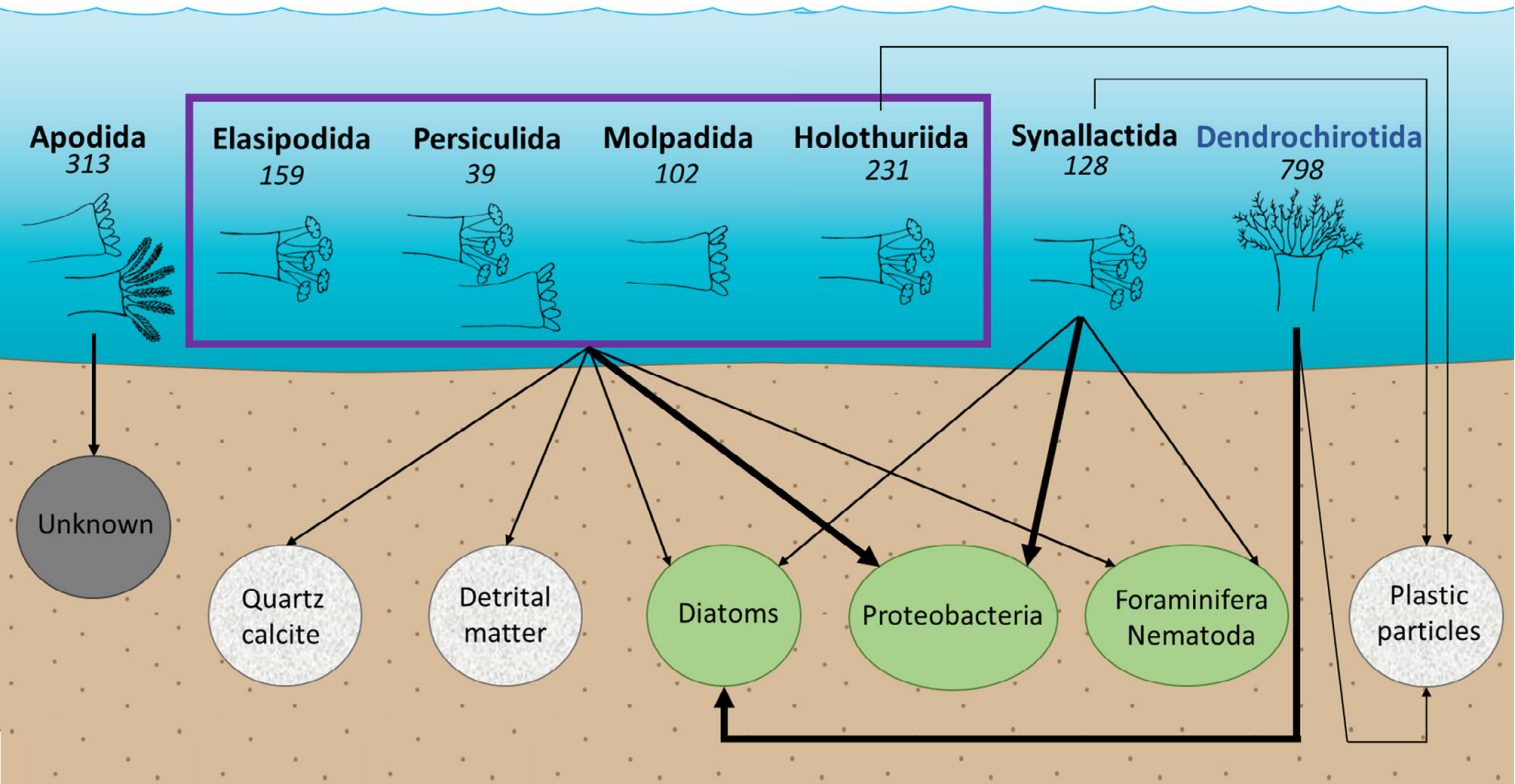
Synthesis of food sources for the seven orders of holothurians. Order colours represent different trophic modes: blue, suspension‐feeders (Dendrochirotida); black, deposit‐feeders. The number of species per taxon is shown below the order name. The width of arrows corresponds to the proportion of food ingested per source. Colours of food sources vary with food type: green, living fraction; grey, non‐living fraction. Tentacle type for each taxon is represented by the drawings (see Fig. 2): digitate (unbranched); peltate (slightly branched); pinnate (highly branched); dendritic (ultra‐branched).

All deposit‐feeding holothurian species studied to date regarding the abundance of bacteria have peltate tentacles, except *M. musculus*, which has digitate tentacles (Amaro *et al*., 2012). No quantitative difference in the ingestion of bacteria has been observed between species with these two tentacle types (Fig. 5). No studies to date have focused on species in Apodida and Dendrochirotida (Table 3). Apodida with pinnate (highly branched) tentacles and suspension‐feeding Dendrochirotida with dendritic (ultra‐branched) tentacles are likely to show different ingestion rates of bacteria due to their higher tentacle surface area or different feeding behaviour.

Our understanding of the profiles of bacterial abundance in holothurian guts is based on only nine species, and 131 individuals, and additional data on the ingestion of bacteria by holothurians are needed.

Deming & Colwell (1982) suggested that holothurians can enhance the value of ingested bacteria by cultivating them in the foregut. Some bacteria may inhabit internal pouches or be attached to the gut epithelium (Harris, 1993). These enteric bacterial strains could help holothurians to digest large molecules through their secretion of hydrolytic ectoenzymes (Roberts *et al*., 1991). Amaro *et al*. (2012) studied bacterial diversity through the digestive tract of *M. musculus*. They found that bacterial diversity showed the same pattern as bacterial biomass: there was a higher number of operational taxonomic units (OTUs) in the foregut (< 100 OTUs) than in sediment (28–71 OTUs), and bacterial diversity decreased towards the hindgut (< 70 OTUs). These observations may highlight the ability of holothurians to cultivate particular strains of bacteria in their foregut. As some endosymbiotic bacterial production could be involved, this requires further investigation.

Only a few studies have explored bacterial diversity in the holothurian gut. Five species have been investigated to date, including one deep‐sea species. All these studies found Proteobacteria to be the most abundant taxon (Fig. 5), representing 43% of bacteria in the diet of *H. leucospilota* (Zhang *et al*., 2012), 86% in *H. scabra* (Plotieau *et al*., 2013), and 55% (Sha *et al*., 2016) and 89.6% (Gao *et al*., 2017) in *A. japonicus*. The forms ingested most were γ‐proteobacteria and α‐proteobacteria for all holothurian species studied. β‐proteobacteria, δ‐proteobacteria and ε‐proteobacteria were also present in some species.

In coastal species, 15 taxa of bacteria were identified, whereas only seven taxa were found in deep‐sea species. Five of these taxa (γ‐proteobacteria, α‐proteobacteria, CFB lineage, Bacteroidetes and Spirochaetes) were shared between coastal and deep‐sea species, perhaps because the diversity of deep‐sea bacteria is lower than the diversity of coastal bacteria.

Knowledge on the abundance and diversity of bacteria is based on the study of less than 500 specimens from only 10 species: four from coastal waters and six from the deep sea belonging to Elasipodida, Persiculida, Holothuriida, Molpadida and Synallactida (Table 3). To the extent that these limited findings allow, it can be concluded that holothurians ingest mainly proteobacteria and show a similar distribution of bacteria through the gut across holothurian species, habitat and tentacle type (Fig. 5).

#### (b) Photosynthetic organisms

Holothurians also consume photosynthetic organisms from both the microphytobenthos (including benthic diatoms, cyanophytes, dinoflagellates, etc.) and macrophytes (macroalgae and phanerogams) for deposit‐feeding holothurians, and phytoplankton (including pelagic diatoms, cyanobacteria, dinoflagellates, etc.) for suspension‐feeding holothurians.

The microphytobenthos in shallow‐water sediments is mainly dominated by diatoms (Uthicke & Klumpp, 1998; Suzumura *et al*., 2002; Heil *et al*., 2004). Taddéi (2006) recorded a mean abundance of 933 diatom cells g^−1^ in the sediment of Reunion Island. In deep‐sea sediments, diatoms are also at surprisingly high concentrations (Wood, 1956; Van Iperen *et al*., 1987; Thiel *et al*., 1989), with high abundances of cyanophytes and dinoflagellates associated with deposited phytodetritus (Lochte & Turley, 1988; Thiel *et al*., 1989).

Nine studies have analysed the importance of photosynthetic organisms in the diet of holothurians (Table 3), of which seven focused on 10 coastal species (Hamel *et al*., 1993; Hamel & Mercier, 1998; Uthicke, 1999; Sonnenholzner, 2003; Taddéi, 2006; Kang *et al*., 2008; Belbachir & Mezali, 2018) and two studies on four deep‐sea species (Khripounoff & Sibuet, 1980; Tyler *et al*., 1992).

The most abundant photosynthetic taxa in the diet of holothurians seems to be diatoms (Fig. 5). Hamel *et al*. (1993) analysed the gut contents of *Psolus fabricii* (Düben & Koren), a suspension‐feeding species, and found fewer than 6000 pelagic diatom cells in the first centimeter of the foregut. Diatoms were recorded at much higher concentrations in the gut of *Holothuria* (*Selenkothuria*) *theeli* Deichmann (up to 16,500 organisms ml^−1^; Sonnenholzner, 2003). For the deep‐sea species investigated, the sighting frequency of diatoms in *P. longicauda*, *P. pallens*, *B. rosea*, and *M. blakei* gut ranges between 51 and 84% (Khripounoff & Sibuet, 1980). Tyler *et al*. (1992) also found a high abundance of diatoms in the gut of *P. pallens*. However, these studies provide no information on whether the diatoms were alive or whether only the siliceous outer skeleton was present.

Photosynthetic organisms may be significant in the diet of holothurians. Fresh organic matter provided by cyanophytes, diatoms, macroalgae and live seagrass leaves represents more than 50% of the diet for *Holothuria poli*, *H. tubulosa*, and *H. forskali* in *Posidonia oceanica* meadows in Algeria (Belbachir & Mezali, 2018). In shallow waters off Ecuador, 35% of the gut content of *H. theeli* consisted of microphytobenthos, with the most dominant taxa being diatoms (91%), followed by cyanophytes and dinoflagellates (Sonnenholzner, 2003). However, these studies did not assess all the possible dietary fractions for holothurians, such as bacteria or minerals, therefore these high proportions may be significant overestimates.

Hamel *et al*. (1993) estimated that phytoplanktonic cells represent between 20 and 50% of the gut content of *P. fabricii* in autumn and winter, increasing during spring to reach 100% in summer in the St. Lawrence Estuary (Canada). They conclude that the ingestion of phytoplanktonic cells, especially pelagic diatoms ingested by this dendrochirotidan holothurian (suspension feeder), depends on seasonal blooms. Similar results were observed for *C. frondosa*, with the proportion of phytoplanktonic cells ingested reaching a maximum in summer (Hamel & Mercier, 1998). Sonnenholzner (2003) investigated the ingestion of photosynthetic organisms in a coastal deposit‐feeding species from Holothuriida, finding that the relative abundance of microphytobenthic species in the *H. theeli* gut was significantly higher during the dry season (45.5%) than in the rainy season (20.2%) in the Gulf of Guayaquil. It would be interesting to extend these findings to all fractions present in the holothurian diet to understand which components replace photosynthetic organisms during the cold seasons.

Taddéi (2006) used the concentration of chlorophyll a as proxy for photosynthetic organisms in the gut for two coastal deposit‐feeders, *H. atra* and *H. leucospilota*. The profile observed was the same as for bacterial abundance: chlorophyll a concentration increased from the local sediment (2.87 ± 1.17 μg g^−1^) to the foregut (18.74 ± 7.65 μg g^−1^), decreased in the hindgut (11.28 ± 4.61 μg g^−1^), and remained at this level in the faeces (11.34 ± 4.63 μg g^−1^). These observations may indicate that photosynthetic organisms are actively selected for ingestion by these deposit‐feeding holothurians.

These studies combined used 378 individuals from six orders of holothurians: Dendrochirotida, Holothuriida, Synallactida, Molpadida, Elasipodida and Persiculida (Table 3). Benthic and pelagic diatoms are a significant dietary component for deposit‐feeding and suspension‐feeding holothurians respectively, independent of habitat, taxon and tentacle type (Fig. 5). However, the importance of photosynthetic organisms remains unclear without further studies because their biomass is subject to seasonal variations.

#### (c) Meiofauna

Meiofauna densities in shallow‐water sediments range from 3.2 to 1,020.6 individuals 10 cm^−2^ (Guzman, Obando & Cortés, 1987; Armenteros, Creagh & González‐Sansón, 2009). In deep‐sea sediments, meiofauna densities are lower than in shallow waters, ranging from 15 to 315 individuals 10 cm^−2^ (Coull *et al*., 1977; Pfannkuche, 1985). In both ecosystems, nematodes and foraminifera are the dominant taxa (Coull *et al*., 1977; Pfannkuche, 1985; Guzman *et al*., 1987; Armenteros *et al*., 2009).

Only seven studies have considered the ingestion of meiofauna by holothurians (Table 3). Nematoda, Copepoda and Foraminifera seem to be the taxa ingested most frequently by holothurians although there are interspecific differences. *Holothuria atra* ingests up to 79% of the meiofauna present in the sediment, with polychaetes the dominant group (Kang *et al*., 2008). Dissection of *H. theeli* individuals showed that 65% of the gut contents consisted of meiofauna from eight taxa, with a dominance of crustaceans (46%) and foraminifera (35.5%) (Sonnenholzner, 2003). Belbachir & Mezali (2018) demonstrated that the percentage contribution of meiofauna in the holothurian diet can differ among locations and species. They studied the diet of four species *H. poli*, *H. tubulosa*, *H. forskali*, and *Holothuria* (*Platyperona*) *sanctori* Dell Chiaje, at two sites (Stidia and Salamandre in Algeria; separated by less than 20 km). At Salamandre, 20% of the diet of *Holothuria sanctori* consisted of crustaceans, whereas in Stidia crustaceans comprised less than 3%. For the three other species, crustaceans represented only 6% of the diet at Salamandre. The contribution of foraminifera ranged between 3.33% for *H. sanctori* and 15% for *H. forskali* at the same site.

For the deep‐sea species *P. longicauda*, *P. pallens*, and *B. rosea*, the sighting frequency of benthic foraminifera reached 100% of the gut content and was 89% for *M. blakei* (Khripounoff & Sibuet, 1980). The sighting frequency of Nematoda and Copepoda was also high for these four species (96–100% and 0–87%, respectively). Tyler *et al*. (1992) recorded abundant foraminifera in the gut of *P. pallens* but no Nematoda nor Copepoda. Note that the sighting frequency is a poor indicator of the actual contribution of meiofauna to the diet.

Uthicke (1999) compared the abundances of Nematoda, Polychaeta and Harpacticoida between the sediment and the midgut content of *S. chloronotus* and *H. atra*. Nematoda were the dominant taxon in the sediments (31.33 ± 10.90 individuals 2 ml^−1^), but they were sparsely represented in the midgut of these two species (between 0.50 ± 0.84 and 0.66 ± 1.03 individuals 2 ml^−1^). Polychaeta were more uncommon in the sediment (9.66 ± 5.31 individuals 2 ml^−1^) and in the midgut of *S. chloronotus* and *H. atra* (0.17 ± 0.41 and 0.01 ± 0.00 individuals 2 ml^−1^ respectively). Uthicke (1999) concluded that meiofauna play a negligible role in the nutrition of these two species due to the extremely low abundance recorded in the midgut. The ingestion of these two meiofaunal taxa appears to be in proportion to their presence in the sediment, suggesting that meiofauna are probably ingested accidentally with detrital matter (Khripounoff & Sibuet, 1980; Billett *et al*., 1988).

One study focused on the ingestion of larval planktonic stages of meiofauna by a suspension‐feeding holothurian. Meiofaunal eggs and embryos accounted for less than 15% of the intestinal content of *C. frondosa* in autumn and winter (Hamel & Mercier, 1998). Maximum zooplankton presence peaked just after breeding of broadcast‐spawning species. The actual contribution of meiofauna to the diet of suspension‐feeding holothurian thus remains largely unknown. As for photosynthetic organisms, the availability of larval planktonic stages of meiofauna varies seasonally and suspension‐feeding holothurians that ingest larval planktonic stages of meiofauna in proportion to their presence in the water column must utilise other resources in the autumn and winter in cold or temperate waters. This implies that meiofauna are not an essential dietary component for holothurians.

The ingestion of meiofauna has been investigated in all holothurian orders except Apodida (Table 3). The meiofauna ingested differs between deposit‐feeding and suspension‐feeding holothurians (Dendrochirotida), because the latter ingest larval planktonic stages of meiofauna. However, for the other orders Foraminifera, Nematoda, Polychaeta and Crustacea are all ingested at a similarly low rate, independent of tentacle or habitat type (Fig. 5).

### (2) Non‐living fractions

#### (a) Detrital matter

Very few studies have considered the detrital matter (Table 3) ingested by coastal and deep‐sea holothurians. Shallow‐water species can feed on detrital matter from seagrasses or algae. Massin & Jangoux (1976) recorded *H. tubulosa* feeding on detrital seagrass leaves and Costa *et al*. (2014) reported a population of *H. tubulosa* to ingest 30–100% of the detritus of *P. oceanica* meadows, depending on holothurian density. Dead *Posidonia* leaves comprise 0.66–14% of the gut content of *H. tubulosa* and *H. forskali*, respectively (Belbachir & Mezali, 2018). Deep‐sea ecosystems also receive phytodetritus, such as dead leaves of seagrasses or algae (Inman & Frautschy, 1965). Using stable isotope analyses, Suchanek *et al*. (1985) reported that at least two deep‐sea species, *Mesothuria verrilli* (Théel) and *Benthodytes linqua* Perrier R., feed on sediments enriched by decaying seagrasses and consume and metabolise seagrass detritus. Khripounoff & Sibuet (1980) estimated that *P. pallens*, *M. blackei*, *B. rosea* and *P. longicauda* feed on macrophytic detritus, although with a low sighting frequency of 13%. Together these observations indicate that detrital matter from macrophytes, in both coastal and deep‐sea species is ingested relatively rarely (Fig. 5).

In the deep sea, only a small fraction of macroaggregates originating from the euphotic zone reaches the seabed to form detrital matter, with a low increment of 100–150 m day^−1^ (Gooday & Turley, 1990), limiting their accessibility for holothurians (Thurston *et al*., 1994; Thurston, Rice & Bett, 1998). Most of this fraction is faecal matter, which represents 95% of vertical particle flow (Wiebe, Boyd & Winget, 1976; Honjo, 1978) and constitutes a key component of the abyssal food web (Frankenberg & Smith, 1967). Holothurians are known to be coprophagous (Bakus, 1973; Hauksson, 1979). However, it remains unclear whether coprophagous holothurians feed on faecal pellets intentionally or simply take advantage of a proximate source of concentrated nutrients. Faecal pellets were found in the gut of all specimens of *P. longicauda*, *P. pallens*, *B. rosea*, and *M. blakei* examined (Khripounoff & Sibuet, 1980). These faecal pellets were from bivalves, pelagic crustaceans, and unidentified sources. Faecal pellets have not been recorded in the diet of coastal species.

Several studies have suggested that detrital matter is a major dietary component for both shallow‐water and deep‐sea holothurian species (Bordovskiy *et al*., 1974; Yingst, 1976; Massin, 1982; Moriarty, 1982; Jeffreys *et al*., 2011). Plotieau (2012) focused on the assimilation of organic compounds from seagrass phytodetritus by *H. scabra*. Experiments showed that *H. scabra* assimilated organic compounds from seagrass leaves, however, this assimilation appeared insufficient to support juvenile growth. Plotieau (2012) therefore suggested that the ingestion of seagrass detritus could be related to the heterotrophic bacteria and microautotrophs attached to them. Indeed, detrital matter, such as phytodetritus, is mainly degraded by bacteria, which colonise it rapidly in both coastal and deep‐sea ecosystems (Fenchel & Jorgensen, 1977; Lochte & Turley, 1988; Thiel *et al*., 1989; Kaiser & Benner, 2008). If correct, this suggests that ingestion of detrital material by holothurians may represent an opportunistic behaviour, to access the bacteria attached to it, rather than detritivorous feeding *per se*.

The ingestion of detrital matter has been relatively poorly studied, with fewer than 100 individuals analysed (Table 3). No studies have focused on Dendrochirotida (suspension feeders) nor Synallactida. Deep‐sea species ingest faecal pellets while coastal species do not. Overall, detrital matter may not be a significant fraction in the diet of holothurians, with the small quantities ingested probably more linked to the presence of bacteria.

#### (b) Minerals

The non‐organic fractions ingested by holothurians are receiving increasing attention, with innovative work suggesting an important role in feeding efficiency and nutritional benefits (Plotieau, 2012).

The sediments ingested by deposit‐feeding holothurians contain insoluble clastic products originating from physical and biological breakdown. An analysis of minerals in the sediment ingested by tropical shallow‐water holothurians identified both primary and secondary minerals (Plotieau, 2012). The primary minerals were (*i*) quartz made up of a continuous framework of SiO_4_ (identified as the main mineral holothurian gut component in most studies), (*ii*) calcite, the most stable polymorph of calcium carbonate (CaCO_3_), (*iii*) aragonite, a thermodynamically unstable form of CaCO_3_ at standard temperature and pressure, and (*iv*) magnesian calcite (Ca, Mg)CO_3_, a variety of calcite containing randomly substituted magnesium carbonate in a disordered calcite lattice that is present in echinoderm skeletons. The secondary minerals were (*i*) bioclasts, skeletal fragments of marine or land organisms found in sedimentary rocks (mainly composed of aragonite but also magnesian calcite and calcite), (*ii*) feldspars (KAlSi_3_O_8_, NaAlSi_3_O_8_, CaAl_2_Si_2_O_8_), a group of rock‐forming tectosilicate minerals, (*iii*) hornblende or dark amphibole, an isomorphous mixture of calcium–iron–magnesium silicate, aluminium–iron–magnesium silicate, and iron–magnesium silicate), and (*iv*) other, trace minerals, mainly zircon.

Three studies analysed the gut contents of shallow‐water or deep‐sea species with respect to minerals (Table 3; Khripounoff & Sibuet, 1980; Tyler *et al*., 1992; Belbachir & Mezali, 2018). They identified the presence of mollusc, echinoderm, and sponge ossicles along with various pelagic and benthic foraminifera tests. The mineral fraction in the diets of four shallow‐water species can exceed 30%, with less than 12% bivalve shells and 12–24% sponge ossicles (Belbachir & Mezali, 2018). A high sighting frequency of 59–79% was reported for spicules and 100% for coccoliths, in the diets of four deep‐sea species (*P. longicauda*, *P. pallens*, *B. rosea*, and *M. blakei*; Khripounoff & Sibuet, 1980). Tyler *et al*. (1992) only rarely recorded spicules or ossicles in the gut of *P. pallens* although coccoliths were abundant.

#### (c) Plastic particles

Recent decades have revealed the impact of increasing plastic input into the ocean on marine taxa, such as fishes, seabirds, turtles, and cetaceans (Cole *et al*., 2011), and how plastic particles penetrate the marine trophic web (Ivar do Sul & Costa, 2014). Species belonging to lower trophic levels can ingest high levels of plastic particles because they do not differentiate between these and their preferred food (Renzi *et al*., 2018).

Several studies have recorded the ingestion of plastic particles (Table 3) in shallow‐water deposit‐feeding species (Graham & Thompson, 2009; Renzi *et al*., 2018; Mohsen *et al*., 2019) and suspension‐feeding species (Graham & Thompson, 2009; Iwalaye *et al*., 2020). *C. frondosa*, *Holothuria* (*Semperothuria*) *cinerascens* (Brandt), *Holothuria grisea* Selenka, *Holothuria floridana* (Pourtalès) and *H. tubulosa* were all shown to ingest plastic particles. Mohsen *et al*. (2019) found a lower abundance of plastic in the gut of farmed *A. japonicus* (0–30 particles per individual) than in the local sediments (20–1040 particles kg^−1^). It is likely that most shallow‐water species will ingest plastics if they are present in the sediment (Graham & Thompson, 2009). Recent studies show that, due to the vertical transport of particles from the sea surface to the sea floor, plastic particles are sequestered in deep‐sea sediments from the Atlantic, Pacific, and Indian Oceans and the Mediterranean Sea (Van Cauwenberghe *et al*., 2013; Woodall *et al*., 2014; Fischer *et al*., 2015). Plastic particles have been found in the gut of other deep‐sea benthic invertebrates including Cnidaria, Echinodermata, Arthropoda (Taylor *et al*., 2016), and Mollusca (Courtene‐Jones *et al*., 2017). Thus, deep‐sea holothurian species may also be exposed to and ingest plastics.

Mohsen *et al*. (2019) also found plastic particles in the coelomic fluid, ranging from 0 to 19 particles per individual. Iwalaye *et al*. (2020) found a similar result in tank experiments with *H. cinerascens* (32–227 microfibres per individual). In more than half of the individuals studied (57.8%), plastic particles were present in the respiratory tree (0–12 microfibres per individual). The biological impacts of the transfer of microplastic particles from the holothurian gut to the rest of the body were not analysed further.

Grossmann (2014) and Assidqi (2015) assessed the impact of the ingestion of plastic particles on *H. sanctori* and *H. leucospilota*, respectively. They exposed these holothurians to plastic particles followed by hypoxic conditions to investigate the effects of plastic particle ingestion on resistance to environmental stress. Ingestion of plastic particles did not affect faeces production, evisceration, respiration rate, survival, or behavioural responses in these two species. However, Assidqi (2015) reported a higher susceptibility of *H. leucospilota* to oxygen depletion 60 days after plastic particle ingestion.

Experiments in mesocosms revealed that holothurians appear preferentially to select plastic particles from the sediment (Graham & Thompson, 2009). The authors hypothesised that the larger surface area of the plastic particles reduces the need to shovel or rake for other particles leading to their selection in preference to sand grains. Similar results were found in a natural environment: analyses of sediment sieved through 63–4,000 μm sieves revealed that *H. tubulosa* selects 100–2,000 μm plastic fragments for ingestion (Renzi *et al*., 2018).

When they reach shallow‐water sediments, plastic particles can rapidly be colonised by bacteria (Harrison *et al*., 2014). Dussud *et al*. (2018) demonstrated that bacterial abundance and diversity is higher on plastic particles than on organic particles. Bacterial communities found on the surface of plastic particles include Proteobacteria, Bacteroidetes (Oberbeckmann *et al*., 2014; Curren & Leong, 2019), and Cyanobacteria (Oberbeckmann *et al*., 2014; Dussud *et al*., 2018). Other organisms, such as diatoms (Carson *et al*., 2013; Eich *et al*., 2015) and dinoflagellates (Masó *et al*., 2003) can also be attached to plastic particles. All of these organisms are found in the holothurian diet. Finally, because holothurians select more bacteria‐rich sediments, they could easily preferentially select plastic particles with bacteria attached, although this requires further investigation.

## V. CONCLUSIONS

1. Cells on the holothurian tentacle bud epidermis are important for food capture. Ciliated cells are likely to be sensory cells that recognise food particles. Mucous cells may be responsible for the production of a physical barrier on the epidermis. The adhesion of food particles is suggested to be performed by the secretion of type‐1 secretory cells; when the tentacles are placed in the oral cavity, adhesion may be removed by the secretion of type‐2 secretory cells. Further studies are needed to confirm these hypothesised roles in holothurian taxa.
2. Holothurian nutrition appears to involve two main feeding modes: some species are suspension‐feeders whereas others are deposit‐feeders, with a few species able to shift between suspension‐ and deposit‐feeding. A third feeding pathway may exist, which relies on nutrient transfer from cloacal water retained by the animal to the haemal system.
3. Synallactida, Molpadida, Persiculida, Holothuriida and Elasipodida, despite their different tentacle types and the different habitats they colonise, ingest similar proportions of different food types. Their diet seems to be composed mainly of proteobacteria. Diatom ingestion may be high, but its overall contribution remains uncertain as its availability varies seasonally. Other smaller fractions, such as detrital matter, minerals and plastic particles, may be ingested only because they support a high bacterial load.
4. Dendrochirotida is the most recent order of holothurians. The species belonging to this order have dendritic tentacles that allow them to act as suspension‐feeders. Most species in Dendrochirotida are coastal (more than 98%). Their trophic specialisation presumably reduces interspecific competition for food, and they ingest large quantities of pelagic diatoms. The ingestion of other food fractions has not yet been studied for Dendrochirotida.
5. There is very little information on the ingestion of food by Apodida. This is a considerable knowledge gap because Apodida represent 17% of holothurian species. More than half of Apodida species have pinnate tentacles that are not present in other orders.

## References

[brv12799-bib-0001] Altamirano, J. P. , Recente, C. P. & Rodriguez, J. C. Jr. (2017). Substrate preference for burying and feeding of sandfish *Holothuria scabra* juveniles. Fisheries Research 186, 514–523.

[brv12799-bib-0002] Amaro, T. , Bianchelli, S. , Billett, D. S. M. , Cunha, M. R. , Pusceddu, A. & Danovaro, R. (2010). The trophic biology of the holothurian *Molpadia musculus* at 3500 m in the Nazaré canyon (NE Atlantic). Biogeosciences Discussions 7, 3061–3094.

[brv12799-bib-0003] Amaro, T. , Luna, G. M. , Danovaro, R. , Billett, D. S. M. & Cunha, M. R. (2012). High prokaryotic biodiversity associated with gut contents of the holothurian *Molpadia musculus* from the Nazaré canyon (NE Atlantic). Deep Sea Research Part I: Oceanographic Research Papers 63, 82–90.

[brv12799-bib-0004] Amaro, T. , Witte, H. , Herndl, G. J. , Cunha, M. R. & Billett, D. S. M. (2009). Deep‐sea bacterial communities in sediments and guts of deposit‐feeding holothurians in Portuguese canyons (NE Atlantic). Deep Sea Research Part I: Oceanographic Research Papers 56, 1834–1843.

[brv12799-bib-0005] Amon, R. M. W. & Herndl, G. J. (1991). Deposit feeding and sediment: I. interrelationship between *Holothuria tubulosa* (Holothurioida, Echinodermata) and the sediment microbial community. Marine Ecology 12, 163–174.

[brv12799-bib-0006] Armenteros, M. , Creagh, B. & González‐Sansón, G. (2009). Distribution patterns of the meiofauna in coral reefs from the NW shelf of Cuba. Revista de Investigaciones Marinas 30(1), 37–43.

[brv12799-bib-0007] Asha, P. S. , Diwakar, K. , Santhanavalli, G. & Manissery, M. K. (2015). Comparative distribution and habitat preference of the sea cucumber *Holothuria atra* jaeger at protected and unprotected sites inThoothukudi region of gulf of Mannar, south‐east coast of India. Indian Journal of Fisheries 62, 52–57.

[brv12799-bib-0008] Assidqi, K. (2015). The Physiological Impact of Microplastics on Holothuria Leucospilota. Bogor Agricultural University, Indonesia.

[brv12799-bib-0009] Bakus, G. J. (1973). The biology and ecology of tropical holothurians. In Biology and Geology of Coral Reefs, Biology I (Volume 11, eds 0. A. Jones and R. Endean ), pp. 325–367. Academic Press, New York.

[brv12799-bib-0010] Baskar, B. K. (1994). Some observations on the biology of the holothurian *Holothuria (Metriatyla) scabra* (jaeger). Bulletin of the Central Marine Fisheries Research Institute 46, 39–43.

[brv12799-bib-0011] Behairy, A. K. A. , Beltagi, S. & Rao, N. V. N. D. (1985). Grain‐size selection and carbonate sediment processing by sea cucumber *Holothuria atra* in the shallow marine environment of Jeddah, eastern Red Sea. Journal of The Faculty of Marine Science 4, 67–77.

[brv12799-bib-0012] Belbachir, N.‐E. & Mezali, K. (2018). Food preferences of four aspidochirotid holothurians species (Holothuroidea: Echinodermata) inhabiting the *Posidonia oceanica* meadow of Mostaganem area (Algeria). SPC Beche‐de mer Information Bulletin 38, 55–59.

[brv12799-bib-0013] Beliaev, G. M. & Brueggeman, P. L. (1989). Deep Sea Ocean Trenches and their Fauna. Nauka, Moskva.

[brv12799-bib-0014] Bell, B. M. (1977). Respiratory schemes in the class Edrioasteroidea. Journal of Paleaontology 51, 619–632.

[brv12799-bib-0015] Billett, D. S. M. , Bett, B. J. , Rice, A. L. , Thurston, M. H. , Galéron, J. , Sibuet, M. & Wolff, G. A. (2001). Long‐term change in the megabenthos of the porcupine abyssal plain (NE Atlantic). Progress in Oceanography 50, 325–348.

[brv12799-bib-0016] Billett, D. S. M. , Lampitt, R. S. , Rice, A. L. & Mantoura, R. F. C. (1983). Seasonal sedimentation of phytoplankton to the deep‐sea benthos. Nature 302, 520–522.

[brv12799-bib-0017] Billett, D.S.M. , Llewellyn, C. & Watson, J. (1988). Are deep‐sea holothurians selective feeders? In: burke et al. (Eds.). *Echinoderm biology: proceedings of the sixth international echinoderm conference*, 421–429.

[brv12799-bib-0018] Boetius, A. & Felbeck, H. (1995). Digestive enzymes in marine invertebrates from hydrothermal vents and other reducing environments. Marine Biology 122, 105–113.

[brv12799-bib-0019] Boon, A. R. & Duineveld, G. C. A. (2012). Phytopigments and fatty acids in the gut of the deposit‐feeding heart urchin *Echinocardium cordatum* in the southern North Sea: selective feeding and its contribution to the benthic carbon budget. Journal of Sea Research 67, 77–84.

[brv12799-bib-0020] Bordovskiy, O. K. , Sokolova, M. N. , Smunov, B. A. , Akhlet'Y, Y. A. & Zezina, O. N. (1974). Evaluation of the role of bottom fauna in the transformation of organic matter in sediments (with specific reference to the deep sea detritus feeders in the Kuril Kamchatka trench). Oceanology 14, 128–132.

[brv12799-bib-0021] Bouland, C. , Massin, C. & Jangoux, M. (1982). The fine structure of the buccal tentacles of *Holothuria forskali* (Echinodermata, Holothuroidea). Zoomorphology 101, 133–149.

[brv12799-bib-0022] Brothers, C. J. , Lee, R. W. & Nestler, J. R. (2015). The uptake of dissolved organic material by the sea cucumber *Parastichopus californicus* (Stimpson) and its potential role in visceral regeneration. Journal of Experimental Marine Biology and Ecology 469, 69–75.

[brv12799-bib-0023] Brown, W. I. & Shick, J. M. (1979). Bimodal gas exchange and the regulation of oxygen uptake in holothurians. The Biological Bulletin 156, 272–288.

[brv12799-bib-0024] Cadée, G. C. (1984). ‘Opportunistic feeding’, a serious pitfall in trophic structure analysis of (paleo) faunas. Lethaia 17, 289–292.

[brv12799-bib-0025] Cameron, J. L. & Fankboner, P. V. (1984). Tentacle structure and feeding processes in life stage of the commercial sea cucumber *Parastichopus californicus* (Smitson). Journal of Experimental Marine Biology and Ecology 81, 193–209.

[brv12799-bib-0026] Carson, H. S. , Nerheim, M. S. , Carroll, K. A. & Eriksen, M. (2013). The plastic‐associated microorganisms of the North Pacific gyre. Marine Pollution Bulletin 75, 126–132. 23993070 10.1016/j.marpolbul.2013.07.054

[brv12799-bib-0027] Choe, S. (1963). Biology of the Japanese Common Sea Cucumber Stichopus Japonicus, Selenka. Pusan National University, Pusan.

[brv12799-bib-0028] Clifford, C. , Walsh, J. , Reidy, N. & Johnson, D. B. (1982). Digestive enzymes and subcellular localization of disaccharidases in some echinoderms. Comparative Biochemistry and Physiology Part B: Comparative Biochemistry 71, 105–110.

[brv12799-bib-0029] Cole, M. , Lindeque, P. , Halsband, C. & Galloway, T. S. (2011). Microplastics as contaminants in the marine environment: a review. Marine Pollution Bulletin 62, 2588–2597. 22001295 10.1016/j.marpolbul.2011.09.025

[brv12799-bib-0030] Conand, C. (1990). The Fishery Resources of Pacific Island Countries: Holothurians. *Food & Agriculture Organization* , Rome.

[brv12799-bib-0031] Conand, C. (2001). Overview of sea cucumbers fisheries over the last decade‐what possibilities for a durable management? Echinoderms 2000, 339–344.

[brv12799-bib-0032] Conand, C. (2008). Population status, fisheries and trade of sea cucumbers in Africa and the Indian Ocean. In: Sea Cucumbers: A Global Review of Fisheries and Trade, Vol. 516. (eds V. Toral‐Granda, A. Lovatelli and M. Vascon‐cellos), Fisheries and Aquaculture Technical Paper No. 516, Rome, pp. 143–193.

[brv12799-bib-0033] Conand, C. (2018). Tropical Sea cucumber fisheries: changes during the last decade. Marine Pollution Bulletin 133, 590–594. 30041353 10.1016/j.marpolbul.2018.05.014

[brv12799-bib-0034] Conde, E. C. , Diaz, H. & Sambrani, A. (1991). Disintegration of holothurian faecal pellets in beds of the seagrass *Thalassia testudinum* . Journal of Coastal Research 7(3), 853–862.

[brv12799-bib-0035] Costa, V. , Mazzola, A. & Vizzini, S. (2014). *Holothuria tubulosa* Gmelin 1791 (Holothuroidea, Echinodermata) enhances organic matter recycling in *Posidonia oceanica* meadows. Journal of Experimental Marine Biology and Ecology 461, 226–232.

[brv12799-bib-0036] Costelloe, J. & Keegan, B. F. (1984). Feeding and related morphological structures in the dendrochirote *Aslia lefevrei* (Holothuroidea: Echinodermata). Marine Biology 84, 135–142.

[brv12799-bib-0037] Coull, B. C. , Ellison, R. L. , Fleeger, J. W. , Higgins, R. P. , Hope, W. D. , Hummon, W. D. , Rieger, R. M. , Sterrer, W. E. , Thiel, H. & Tietjen, J. H. (1977). Quantitative estimates of the meiofauna from the deep sea off North Carolina, USA. Marine Biology 39, 233–240.

[brv12799-bib-0038] Coulon, P. & Jangoux, M. (1993). Feeding rate and sediment reworking by the holothuroid *Holothuria tubulosa* (Echinodermata) in a Mediterranean seagrass bed off Ischia Island, Italy. Marine Ecology Progress Series 92, 201–204.

[brv12799-bib-0039] Courtene‐Jones, W. , Quinn, B. , Gary, S. F. , Mogg, A. O. M. & Narayanaswamy, B. E. (2017). Microplastic pollution identified in deep‐sea water and ingested by benthic invertebrates in the Rockall trough, North Atlantic Ocean. Environmental Pollution 231, 271–280. 28806692 10.1016/j.envpol.2017.08.026

[brv12799-bib-0040] Cuénot, L. (1948). Les Echinodermes. Traité de Zoologie, p. 363. Masson et Cie, Grasse.

[brv12799-bib-0041] Cui, L. B. , Dong, Z. N. & Lu, Y. H. (2000). Histological and histochemical studies on the digestive system of *Apostichopus japonicus* . Chinese Journal of Zoology 35, 1–4.

[brv12799-bib-0042] Curren, E. & Leong, S. C. Y. (2019). Profiles of bacterial assemblages from microplastics of tropical coastal environments. Science of the Total Environment 655, 313–320. 30471599 10.1016/j.scitotenv.2018.11.250

[brv12799-bib-0043] Cushing, D. H. (1959). On the nature of production in the sea. Fisheries Investigations. London 22, 1–40.

[brv12799-bib-0044] Cuvillier, A . (2016). *Dynamique et fonctionnement des herbiers marins dans un complexe récifal anthropisé (île de la Réunion*, *océan Indien)*. PhD Thesis: La Réunion.

[brv12799-bib-0045] Da Silva, J. , Cameron, J. L. & Fankboner, P. V. (1986). Movement and orientation patterns in the commercial sea cucumber *Parastichopus californicus* (Stimpson) (Holothuroidea Aspidochirotida). Marine Bahaviour Physiology 12, 133–147.

[brv12799-bib-0046] Dale, N. G. (1974). Bacteria in intertidal sediments: factors related to their distribution: Bacteria in sediments. Limnology and Oceanography 19, 509–518.

[brv12799-bib-0047] Danovaro, R. , Fabiano, M. & Della Croce, N. (1993). Labile organic matter and microbial biomasses in deep‐sea sediments (Eastern Mediterranean Sea). Deep Sea Research Part I: Oceanographic Research Papers 40, 953–965.

[brv12799-bib-0048] Danovaro, R. , Marrale, D. , Croce, N. D. , Dell'Anno, A. & Fabiano, M. (1998). Heterotrophic Nanoflagellates, Bacteria, and labile organic compounds in continental shelf and Deep‐Sea sediments of the eastern Mediterranean. Microbial Ecology 35, 244–255. 9569282 10.1007/s002489900080

[brv12799-bib-0049] Dar, M. A. (2004). Holothurian role in the marine sediments reworking processes. Sedimentology of Egypt 12, 173–183.

[brv12799-bib-0050] Dar, M. A. & Ahmad, H. O. (2006). The feeding selectivity and ecological role of shallow water holothurians in the Red Sea. SPC Beche‐de mer Information Bulletin 24, 11–21.

[brv12799-bib-0051] De Leo, F. C. , Smith, C. R. , Rowden, A. A. , Bowden, D. A. & Clark, M. R. (2010). Submarine canyons: hotspots of benthic biomass and productivity in the deep sea. Proceedings of the Royal Society B: Biological Sciences 277, 2783–2792. 10.1098/rspb.2010.0462 PMC2981985 20444722

[brv12799-bib-0052] Deming, J. W. & Colwell, R. R. (1982). Barophilic Bacteria associated with digestive tracts of abyssal holothurians. Applied and Environmental Microbiology 44, 1222–1230. 16346137 10.1128/aem.44.5.1222-1230.1982 PMC242171

[brv12799-bib-0053] Di Simone, M. , Horellou, A. & Conand, C. (2019). Towards a CITES listing of teatfish. SPC Beche‐de‐mer Information Bulletin 39, 76–78.

[brv12799-bib-0054] Dissanayake, D. C. T. & Stefansson, G. (2010). Abundance and distribution of commercial sea cucumber species in the coastal waters of Sri Lanka. Aquatic Living Resources 23, 303–313.

[brv12799-bib-0055] Dolmatov, I. Y. & Ginanova, T. T. (2009). Post‐autotomy regeneration of respiratory trees in the holothurian *Apostichopus japonicus* (Holothuroidea, Aspidochirotida). Cell and Tissue Research 336, 41–58. 19238446 10.1007/s00441-009-0761-6

[brv12799-bib-0056] Dorsett, D. A. & Hyde, R. (1969). The fine structure of the compound sense organs on the cirri of *Nereis diversicolor* . Zeitschrift für Zellforschung und Mikroskopische Anatomie 97, 512–527. 4907385 10.1007/BF00332800

[brv12799-bib-0057] Dussud, C. , Meistertzheim, A. L. , Conan, P. , Pujo‐Pay, M. , George, M. , Fabre, P. , Coudane, J. , Higgs, P. , Elineau, A. , Pedrotti, M. L. , Gorsky, G. & Ghiglione, J. F. (2018). Evidence of niche partitioning among bacteria living on plastics, organic particles and surrounding seawaters. Environmental Pollution 236, 807–816. 29459335 10.1016/j.envpol.2017.12.027

[brv12799-bib-0058] Eich, A. , Mildenberger, T. , Laforsch, C. & Weber, M. (2015). Biofilm and diatom succession on polyethylene (PE) and biodegradable plastic bags in two marine habitats: early signs of degradation in the pelagic and benthic zone? PLoS One 10, e0137201. 26394047 10.1371/journal.pone.0137201 PMC4578875

[brv12799-bib-0059] Fankboner, P. V. (1978). Suspension‐feeding mechanisms of the armoured sea cucumber *Psolus chitinoides* (Clark). Journal of Experimental Marine Biology and Ecology 31, 11–25.

[brv12799-bib-0060] Fankboner, P. V. (1981). A re‐examination of mucus feeding by the sea cucumber *Leptopentacta* (*Cucumaria*) *elongata* . Journal of the Marine Biological Association of the United Kingdom 61, 679–683.

[brv12799-bib-0061] Fenchel, T. M. & Jorgensen, B. B. (1977). Detritus food chains of aquatic ecosystems: the role of Bacteria. In Advances in Microbial Ecology (ed. M. Alexander ), pp. 1–58. Plenum Press, New York.

[brv12799-bib-0062] Féral, J. F. & Massin, C. (1982). Digestive systems: Holothuroidea. In Echinoderm Nutrition (eds M. Jangoux and J. M. Lawrence ), pp. 191–212. AA Balkema, Rotterdam.

[brv12799-bib-0063] Féral, J.‐P. (1989). Activity of the principal digestive enzymes in the detritivorous apodous holothuroid *Leptosynapta galliennei* and two other shallow‐water holothuroids. Marine Biology 101, 367–379.

[brv12799-bib-0064] Féral, J.‐P. & Magniez, P. (1985). Level, content and energetic equivalent of the main biochemical constituents of the subantarctic molpadid holothurian *Eumolpadia violacea* (echinodermata) at two seasons of the year. Comparative Biochemistry and Physiology Part A: Physiology 81, 415–422.

[brv12799-bib-0065] Fischer, V. , Elsner, N. O. , Brenke, N. , Schwabe, E. & Brandt, A. (2015). Plastic pollution of the Kuril–Kamchatka trench area (NW pacific). Deep Sea Research Part II: Tropical Studies in Oceanography 111, 399–405.

[brv12799-bib-0066] Fish, J. D. (1967). The digestive system of the holothurian *Cucumaria elongata*. II. Distribution of digestive enzymes. The biological Bulletin 132, 354–361.

[brv12799-bib-0067] Flammang, P. & Conand, C. (2004). Functional morphology of the tentacles in the apodid holothuroid *Synapta maculata*. Echinoderms, pp. 327–332.

[brv12799-bib-0068] Fontaine, A. R. & Chia, F. S. (1968). Echinoderms: an autoradiographic study of assimilation of dissolved organic molecules. Science 161, 1153–1155. 17812294 10.1126/science.161.3846.1153

[brv12799-bib-0069] Fox, H. M. (1952). Anal and oral intake of water by Crustacea. Journal of Experimental Biology 29, 583–599.

[brv12799-bib-0070] Frankenberg, D. & Smith, K. L. (1967). Coprophagy in marine animals. Limnology and Oceanography 12, 443–450.

[brv12799-bib-0071] Friedman, K. , Eriksson, H. , Tardy, E. & Pakoa, K. (2011). Management of sea cucumber stocks: patterns of vulnerability and recovery of sea cucumber stocks impacted by fishing. Fish and Fisheries 12, 75–93.

[brv12799-bib-0072] Gao, M. L. , Hou, H. M. , Zhang, G. L. , Liu, Y. & Sun, L. M. (2017). Bacterial diversity in the intestine of sea cucumber *Stichopus japonicus* . Iranian Journal of Fisheries Sciences 16, 318–325.

[brv12799-bib-0073] Ginger, M. L. , Billett, D. S. M. , Mackenzie, K. L. , Kiriakoulakis, K. , Neto, R. R. , Boardman, D. K. , Santos, V. L. C. S. , Horsfall, I. M. & Wolff, G. A. (2001). Organic matter assimilation and selective feeding by holothurians in the deep sea: some observations and comments. Progress in Oceanography 50, 407–421.

[brv12799-bib-0074] Gooday, A. J. & Turley, C. M. (1990). Responses by benthic organisms to inputs of organic material to the ocean floor: a review. Philosophical Transactions of the Royal Society of London. Series A, Mathematical and Physical Sciences 331, 119–138.

[brv12799-bib-0075] Graham, E. R. & Thompson, J. T. (2009). Deposit‐ and suspension‐feeding sea cucumbers (Echinodermata) ingest plastic fragments. Journal of Experimental Marine Biology and Ecology 368, 22–29.

[brv12799-bib-0076] Grossmann, J.L. (2014). *Evaluating the Potential of Microplastics Ingestion to Harm the Sea* Cucumber *Holothuria Sanctori*. PhD Thesis: University of Hamburg, pp. 100.

[brv12799-bib-0077] Gutt, J. (1990). New Antarctic holothurians (Echinodermata) ‐ I. five new species with four new genera of the order Dendrochirotida. Zoologica Scripta 19, 101–117.

[brv12799-bib-0078] Guzman, H. M. , Obando, V. L. & Cortés, J. (1987). Meiofauna associated with a Pacific coral reef in Costa Rica. Coral Reefs 6, 107–112.

[brv12799-bib-0079] Hamel, J. F. , Himmelman, J. H. & Dufresne, L. (1993). Gametogenesis and spawning of the sea cucumber *Psolus fabricii* (Duben and Koren). The Biological Bulletin 184, 125–143. 29300522 10.2307/1542223

[brv12799-bib-0080] Hamel, J.‐F. & Mercier, A. (1998). Diet and feeding behaviour of the sea cucumber *Cucumaria frondosa* in the St. Lawrence estuary, eastern Canada. Canadian Journal of Zoology 76, 1194–1198.

[brv12799-bib-0081] Hamel, J.‐F. , Sun, J. , Gianasi, B. L. , Montgomery, E. M. , Kenchington, E. L. , Burel, B. , Rowe, S. , Winger, P. D. & Mercier, A. (2019). Active buoyancy adjustment increases dispersal potential in benthic marine animals. Journal of Animal Ecology 88, 820–832. 30636040 10.1111/1365-2656.12943 PMC6850204

[brv12799-bib-0082] Hammond, L. (1983). Nutrition of deposit‐feeding holothuroids and echinoids (Echinodermata) from a shallow reef lagoon, Discovery Bay, Jamaica. Marine Ecology Progress Series 10, 297–305.

[brv12799-bib-0083] Hammond, L. S. (1982). Patterns of feeding and activity in deposit‐feeding holothurians and echinoids (Echinodermata) from a shallow back‐reef lagoon, Discovery Bay, Jamaica. Bulletin of Marine Science 32, 549–571.

[brv12799-bib-0084] Hammond, L. S. & Wilkinson, C. R. (1985). Exploitation of sponge exudates by coral reef holothuroids. Journal of Experimental Marine Biology and Ecology 94, 1–9.

[brv12799-bib-0085] Hansen, B. (1975). Systematics and Biology of the Deep‐Sea Holothurians: Elasipoda. Scandinavian Science Press, Scandinavia.

[brv12799-bib-0086] Harris, J. M. (1993). The presence, nature, and role of gut microflora in aquatic invertebrates: a synthesis. Microbial Ecology 25, 195–321. 24189919 10.1007/BF00171889

[brv12799-bib-0087] Harrison, J. P. , Schratzberger, M. , Sapp, M. & Osborn, A. M. (2014). Rapid bacterial colonization of low‐density polyethylene microplastics in coastal sediment microcosms. BMC Microbiology 14, 232. 25245856 10.1186/s12866-014-0232-4 PMC4177575

[brv12799-bib-0088] Hauksson, E. (1979). Feeding biology of *Stichopus tremulus*, a deposit‐ feeding holothurian. Sarsia 64, 155–160.

[brv12799-bib-0089] Heil, C. A. , Chaston, K. , Jones, A. , Bird, P. , Longstaff, B. , Costanzo, S. & Dennison, W. C. (2004). Benthic microalgae in coral reef sediments of the southern great barrier reef. Coral Reefs 23, 336–343.

[brv12799-bib-0090] Holtz, E. H. & MacDonald, B. A. (2009). Feeding behaviour of the sea cucumber *Cucumaria frondosa* (Echinodermata: Holothuroidea) in the laboratory and the field: relationships between tentacle insertion rate, flow speed, and ingestion. Marine Biology 156, 1389–1398.

[brv12799-bib-0091] Honjo, S. (1978). Sedimentation of materials in the Sargasso Sea at a 5367 m deep station. Journal of Marine Research 36, 469–492.

[brv12799-bib-0092] Hudson, I. R. , Wigham, B. D. , Solan, M. & Rosenberg, R. (2005). Feeding behaviour of deep‐sea dwelling holothurians: inferences from a laboratory investigation of shallow fjordic species. Journal of Marine Systems 57, 201–218.

[brv12799-bib-0093] Hylleberg, J. & Gallucci, V. F. (1975). Selectivity in feeding by the deposit‐feeding bivalve *Macoma nasuta* . Marine Biology 32, 167–178.

[brv12799-bib-0094] Hyman, L. H. (1955). The invertebrates: echinodermata. The coelomate bilateria 4, 1–763.

[brv12799-bib-0095] Iken, K. , Brey, T. , Wand, U. , Voigt, J. & Junghans, P. (2001). Food web structure of the benthic community at the porcupine abyssal plain (NE Atlantic): a stable isotope analysis. Progress in Oceanography 50, 383–405.

[brv12799-bib-0096] Inman, D.L. & Frautschy, J.D. (1965). Littoral processes and the development of shorelines. In Proceedings Santa Barbara Specialty Conference. ASCE

[brv12799-bib-0097] Ivar do Sul, J. A. & Costa, M. F. (2014). The present and future of microplastic pollution in the marine environment. Environmental Pollution 185, 352–364. 24275078 10.1016/j.envpol.2013.10.036

[brv12799-bib-0098] Iwalaye, O. A. , Moodley, G. K. & Robertson‐Andersson, D. V. (2020). The possible routes of microplastics uptake in sea cucumber *Holothuria cinerascens* (Brandt, 1835). Environmental Pollution 264, 1–9. 10.1016/j.envpol.2020.114644 32559857

[brv12799-bib-0099] Jaeckle, W. B. & Strathmann, R. R. (2013). The anus as a second mouth: anal suspension feeding by an oral deposit‐feeding sea cucumber. Invertebrate Biology 132, 62–68.

[brv12799-bib-0100] James, D. B. & James, P. S. B. R. (1994). A handbook on Indian sea‐ cucumbers. CMFRI Special Publication 59, 1–47.

[brv12799-bib-0101] Jamieson, A. J. , Gebruk, A. , Fujii, T. & Solan, M. (2011). Functional effects of the hadal sea cucumber *Elpidia atakama* (Echinodermata: Holothuroidea, Elasipodida) reflect small‐scale patterns of resource availability. Marine Biology 158, 2695–2703.

[brv12799-bib-0102] Jangoux, M. & Lawrence, J. M. (1982). Echinoderm Nutrition. CRC Press, Boca Raton, Florida.

[brv12799-bib-0103] Jaquemet, S. , Rousset, V. & Conand, C. (1999). Asexual reproduction parameters and the influence of fission on a *Holothuria atra* sea cucumber population from a fringing reef on Reunion Island (Indian Ocean). SPC Beche de Mer Information Bulletin 11, 12–18.

[brv12799-bib-0104] Jeffreys, R. M. , Lavaleye, M. S. S. , Bergman, M. J. N. , Duineveld, G. C. A. & Witbaard, R. (2011). Do abyssal scavengers use phytodetritus as a food resource? Video and biochemical evidence from the Atlantic and Mediterranean. Deep Sea Research Part I: Oceanographic Research Papers 58, 415–428.

[brv12799-bib-0105] Jimmy, R. A. , Pickering, T. D. & Hair, C. A. (eds) (2012). Overview of sea cucumber aquaculture and stocking research in the Western Pacific region. Asia‐Pacific Tropical Sea Cucumber Aquaculture. ACIAR Proceedings 136, 12–21.

[brv12799-bib-0106] Johnstone, R. N. , Koop, K. & Larkum, A. W. D. (1990). Physical aspects of coral reef laqoon sediments in relation to detritus pro‐cessing and primary production. Marine Ecology Progress Series 66, 273–283.

[brv12799-bib-0107] Jumars, P. A. (1993). Gourmands of mud: diet selection in marine deposit feeders. In Diet Selection: An Interdisciplinary Approach to Foraging Behaviour (ed. R. Hughes ), pp. 124–156. Blackwell Scientific, Oxford.

[brv12799-bib-0108] Jumars, P. A. , Self, R. F. L. & Nowell, A. R. M. (1982). Mechanics of particle selection by tentaculate deposit‐feeders. Journal of Experimental Marine Biology and Ecology 64, 47–70.

[brv12799-bib-0109] Kaiser, K. & Benner, R. (2008). Major bacterial contribution to the ocean reservoir of detrital organic carbon and nitrogen. Limnology and Oceanography 53, 99–112.

[brv12799-bib-0110] Kang, D.‐H. , Affan, M.‐A. , Rho, H. S. , Paik, S.‐G. & Park, H.‐S. (2008). Natural feeding of coral reef holothurian, *Holothuria atra* on microalgae and meiofauna from seagrass beds in Chuuk, FSM. SPC Beche‐de‐mer Information Bulletin 28, 57.

[brv12799-bib-0111] Kato, A. & Hirata, H. (1990). Effects of water temperature on the circadian rhythm of the sea‐cucumber, *Stichopus japonicus* in culture. Aquaculture Science 38, 75–80.

[brv12799-bib-0112] Khripounoff, A. & Sibuet, M. (1980). La nutrition d'echinodermes abyssaux I. Alimentation des holothuries. Marine Biology 60, 17–26.

[brv12799-bib-0113] Kinch, J. , Purcell, S. , Uthicke, S. & Friedman, K. (2008). Population status, fisheries and trade of sea cucumbers in the Western Central Pacific. In Sea Cucumbers. A Global Review of Fisheries and Trade. (eds V. Toral‐Granda , A. Lovatelli and M. Vasconcellos ), Vol. 5, pp. 7–55. FAO Fisheries and Aquaculture, Rome.

[brv12799-bib-0114] Kuhnz, L. A. , Ruhl, H. A. , Huffard, C. L. & Smith, K. L. (2014). Rapid changes and long‐term cycles in the benthic megafaunal community observed over 24years in the abyssal Northeast Pacific. Progress in Oceanography 124, 1–11.

[brv12799-bib-0115] Larson, B. R. , Vadas, R. L. & Keser, M. (1980). Feeding and nutritional ecology of the sea urchin *Strongylocentrotus droebachiensis* in Maine, USA. Marine Biology 59, 49–62.

[brv12799-bib-0116] Laverack, M. S. (1974). The structure and function of chemoreceptor cells. In Chemoreception in Marine Organisms (eds P. T. Grant and A. M. Mackie ), pp. 1–48. Academic Press, London; New York.

[brv12799-bib-0117] Lavitra, T. , Rasolofonirina, R. , Jangoux, M. & Eeckhaut, I. (2009). Problems related to the farming of *Holothuria scabra* (jaeger, 1833). SPC Beche‐de mer Information Bulletin 29, 20–31.

[brv12799-bib-0118] Lawrence, J. M. (1982). Digestion: post‐metamorphic and larval echinoderms. In Echinordem nutrition (eds M. Jangoux and J. M. Lawrence ), pp. 283–316. Balkema, Rotterdam.

[brv12799-bib-0119] Lawrence, J. M. & Guille, A. (1982). Organic composition of tropical, polar and temperate‐water echinoderms. Comparative Biochemistry and Physiology Part B: Comparative Biochemistry 72, 283–287.

[brv12799-bib-0120] Lee, S. , Ferse, S.C. , Ford, A. , Wild, C. & Mangubhai, S. (2017). Effect of sea cucumber density on the health of reef‐flat sediments. Wildlife Conservation Society, pp. 54–61.

[brv12799-bib-0121] Levin, V. S. (1982). Japanese Sea Cucumber. Dalizdat, Vladivostok.

[brv12799-bib-0122] Levin, V. S. (1989). Trophoecology of Holothurians of the Nearshore Sea Zone. DSc Thesis, Moscow, P.P, p. 324. Shirshov Institute of Oceanology, Moscow.

[brv12799-bib-0123] Li, F. , Liu, Y. , Song, B. , Sun, H. , Gu, B. & Zhang, X. (1996). Study on aestivating habit of sea cucumber (*Apostichopus japonicus*, Selenka): the factors relating to aestivation. Journal of Fisheries Sciences China 3, 49–57.

[brv12799-bib-0124] Liu, Y. , Li, F. , Song, B. , Sun, H. , Zhang, X. & Gu, B. (1996). Study on aestivating habit of sea cucumber *Apostichopus japonicus* Selenka: ecological characteristic of aestivation. Journal of Fisheries Sciences China 3, 41–48.

[brv12799-bib-0125] Lochte, K. & Turley, C. M. (1988). Bacteria and cyanobacteria associated with phytodetritus in the deep sea. Nature 333, 67–69.

[brv12799-bib-0127] Lopez, G. R. & Levinton, J. S. (1987). Ecology of deposit‐feeding animals in marine sediments. The Quarterly Review of Biology 62, 235–260.

[brv12799-bib-0128] Lovatelli, A. C. C. , Purcell, S. , Uthicke, S. , Hamel, J. F. & Mercier, A. (2004). Advances in Sea Cucumber Aquaculture and Management. Food and Agriculture Organization of the United Nations, Rome.

[brv12799-bib-0129] MacTavish, T. , Stenton‐Dozey, J. , Vopel, K. & Savage, C. (2012). Deposit‐Feeding Sea cucumbers enhance mineralization and nutrient cycling in organically‐enriched coastal sediments. PLoS One 7, e50031. 23209636 10.1371/journal.pone.0050031 PMC3507890

[brv12799-bib-0130] Manship, B.A.D. (1995). *The Feeding Ecology of Deposit‐Feeding Holothurians*. PhD Thesis: Queen's University, Belfast.

[brv12799-bib-0131] Marquet, N. , Hubbard, P. C. , da Silva, J. P. , Afonso, J. & Canário, A. V. M. (2018). Chemicals released by male sea cucumber mediate aggregation and spawning behaviours. Scientific Reports 8, 239. 29321586 10.1038/s41598-017-18655-6 PMC5762768

[brv12799-bib-0132] Masó, M. , Garcés, E. , Pagès, F. & Camp, J. (2003). Drifting plastic debris as a potential vector for dispersing harmful algal bloom (HAB) species. Scientia Marina 67, 107–111.

[brv12799-bib-0133] Massin, C. (1978). Etude de la nutrition chez les holothuries aspidochirotes (Echinodermes). Comportement alimentaire, structure et fonctions de l'appareil digestif. Université libre de Bruxelles, Laboratoire de Zoologie, Bruxelles.

[brv12799-bib-0134] Massin, C. (1982). Food and feeding mechanism: Holothuroidea. In Echinoderm Nutrition (eds M. Jangoux and J. M. Lawrence ), pp. 43–55. A. A. Balkema, Rotterdam.

[brv12799-bib-0135] Massin, C. (1984). Structures digestives d'holothuries Elasipoda (Echinodermata): *Benthogone rosea* Koehler, 1896 et *Oneirophanta mutabilis* Théel, 1879. Archives de Biologie 95, 153–185.

[brv12799-bib-0136] Massin, C. & Doumen, C. (1986). Distribution and feeding of epibenthic holothuroids on the reef flat of Laing Island (Papua New Guinea). Marine Ecology Progress Series 31, 185–195.

[brv12799-bib-0137] Massin, C. & Jangoux, M. (1976). Observations écologiques sur *Holothuria tubulosa*, *H. poli* et *H. forskali* (Echinodermata: Holothuroidea) et comportement alimentaire de *H. tubulosa* . Biology Marine 17, 45–59.

[brv12799-bib-0138] McEdward, L. R. & Miner, B. G. (2001). Larval and life‐cycle patterns in echinoderms. Canadian Journal of Zoology 79, 1125–1170.

[brv12799-bib-0139] McKenzie, J. D. (1987). The ultrastructure of the tentacles of eleven species of dendrochirote holothurians studied with special reference to the surface coats and papillae. Cell and Tissue Research 248, 187–199.

[brv12799-bib-0140] Mellin, C. , Lugrin, C. , Okaji, K. , Francis, D. & Uthicke, S. (2017). Selective feeding and microalgal consumption rates by crown‐of‐thorns seastar (*Acanthaster cf. solaris*) larvae. Diversity 9, 8.

[brv12799-bib-0141] Meng, X. , Dong, Y. , Dong, S. , Yu, S. & Zhou, X. (2011). Mortality of the sea cucumber, *Apostichopus japonicus* Selenka, exposed to acute salinity decrease and related physiological responses: osmoregulation and heat shock protein expression. Aquaculture 316, 88–92.

[brv12799-bib-0142] Mercier, A. , Battaglene, S. C. & Hamel, J.‐F. (1999). Daily burrowing cycle and feeding activity of juvenile sea cucumbers *Holothuria scabra* in response to environmental factors. Journal of Experimental Marine Biology and Ecology 239, 125–156. 10.1016/s0022-0981(00)00187-8 10817830

[brv12799-bib-0143] Mercier, A. , Battaglene, S. C. & Hamel, J.‐F. (2000). Periodic movement, recruitment and size‐related distribution of the sea cucumber *Holothuria scabra* in Solomon Islands. In Island, Ocean and Deep‐Sea Biology (eds M. B. Jones , J. M. N. Azevedo , A. I. Neto , A. C. Costa and A. M. F. Martins ), pp. 81–100. Springer Netherlands, Dordrecht.

[brv12799-bib-0144] Mezali, K. & Soualili, D. L. (2013). The ability of holothurians to select sediment particles and organic matter. SPC Beche‐de mer Information Bulletin 33, 38–43.

[brv12799-bib-0145] Michio, K. , Kengo, K. , Yasunori, K. , Hitoshi, M. , Takayuki, Y. , Hideaki, Y. & Hiroshi, S. (2003). Effects of deposit feeder *Stichopus japonicus* on algal bloom and organic matter contents of bottom sediments of the enclosed sea. Marine Pollution Bulletin 47, 118–125. 12787607 10.1016/S0025-326X(02)00411-3

[brv12799-bib-0146] Miller, A. K. , Kerr, A. M. , Paulay, G. , Reich, M. , Wilson, N. G. , Carvajal, J. I. & Rouse, G. W. (2017). Molecular phylogeny of extant Holothuroidea (Echinodermata). Molecular Phylogenetics and Evolution 111, 110–131. 28263876 10.1016/j.ympev.2017.02.014

[brv12799-bib-0147] Miller, R. J. , Smith, C. R. , Demaster, D. J. & Fornes, W. L. (2000). Feeding selectivity and rapid particle processing by deep‐sea megafaunal deposit feeders: a ^234^Th tracer approach. Journal of Marine Research 58, 653–673.

[brv12799-bib-0148] Mohsen, M. , Wang, Q. , Zhang, L. , Sun, L. , Lin, C. & Yang, H. (2019). Microplastic ingestion by the farmed sea cucumber *Apostichopus japonicus* in China. Environmental Pollution 245, 1071–1078. 30682741 10.1016/j.envpol.2018.11.083

[brv12799-bib-0149] Moriarty, J. W. (1982). Feeding of *Holothuria atra* and *Stichopus chloronotus* on bacteria, organic carbon and organic nitrogen in sediments of the great barrier reef. Australian Journal of Marine and Freshwater Research 33, 255–263.

[brv12799-bib-0150] Mosher, C. (1980). Distributionof *Holothuria arenicola* semper in The Bahamas with observations on habitat, behavior, and feeding activity (Echinodermata: Holothuroidea). Bulletin of Marine Science 30, 1–12.

[brv12799-bib-0151] Myers, A. C. (1977). Sediment processing in a marine subtidal sandy bottom community: I. Physical aspects. Journal of Marine Research 35, 609–632.

[brv12799-bib-0152] Navarro, P. G. , García‐Sanz, S. , Barrio, J. M. & Tuya, F. (2013). Feeding and movement patterns of the sea cucumber *Holothuria sanctori* . Marine Biology 160, 2957–2966.

[brv12799-bib-0153] Nelson, B. V. & Vance, R. R. (1979). Diel foraging patterns of the sea urchin *Centrostephanus coronalus* as a predator avoidance strategy. Marine Biology 51, 251–258.

[brv12799-bib-0154] Newell, R. C. & Courtney, W. A. M. (1965). Respiratory movements in *Holothuria forskali* Delle chiaje. Journal of Experimental Biology 42, 45–57.

[brv12799-bib-0155] Oberbeckmann, S. , Loeder, M. G. J. , Gerdts, G. & Osborn, A. M. (2014). Spatial and seasonal variation in diversity and structure of microbial biofilms on marine plastics in northern European waters. FEMS Microbiology Ecology 90, 478–492. 25109340 10.1111/1574-6941.12409

[brv12799-bib-0156] O'Loughlin, P. M. , Bardsley, T. M. & O'Hara, T. D. A. (2020). A preliminary analysis of diversity and distribution of Holothurioidea from Prydz Bay and the MacRobertson shelf, eastern Antarctica. In Echinoderms Through Time (eds A. Guille , B. David and J.‐P. Feral ), pp. 549–555. Press, CRC.

[brv12799-bib-0157] Paltzat, D. L. , Pearce, C. M. , Barnes, P. A. & McKinley, R. S. (2008). Growth and production of California Sea cucumbers (*Parastichopus californicus* Stimpson) co‐cultured with suspended Pacific oysters (*Crassostrea gigas* Thunberg). Aquaculture 275, 124–137.

[brv12799-bib-0158] Pawson, D. L. (1970). The Marine Fauna of New Zealand: Sea Cucumbers (Echinodermata: Holothuroidea). New Zealand: *Governement Print* .

[brv12799-bib-0159] Pawson, D. L. & Fell, H. B. (1965). A revised classification of the dendrochirote holothurians. Breviora 214, 1–7.

[brv12799-bib-0160] Pearson, J. (1914). Proposed reclasification of the genera Muelleria and Holothuria. Spolia Zeylanica 9, 163–172.

[brv12799-bib-0161] Petch, D. A. (1986). Selective deposit‐feeding by *Lumbrineris cf. latreii* (Polychaeta: Lumbrineridae), with a new method for assessing selectivity by deposit‐feeding organisms. Marine Biology 93, 443–448.

[brv12799-bib-0162] Pfannkuche, O. (1985). The deep‐sea meiofauna of the porcupine Seabight and abyssal plain (NE Atlantic): population structure, distribution, standing stocks. Oceanologica Acta 8, 343–353.

[brv12799-bib-0163] Pitt, R. & Duy, N. D. Q. (2004). Breeding and rearing of the sea cucumber *Holothuria scabra* in Viet Nam. Aquaculture Advances 3, 333–346.

[brv12799-bib-0164] Plotieau, T. (2012). *Analyses de certains éléments nutritionels essentiels à Holothuria scabra (Echinodermata*, *Holothuroidea): influence de la qualité du sédiment sur le développement des holothuries en aquaculture et importance des bactéries*. PhD Thesis: University of Mons, Belgium.

[brv12799-bib-0165] Plotieau, T. , Lavitra, T. , Gillan, D. C. & Eeckhaut, I. (2013). Bacterial diversity of the sediments transiting through the gut of *Holothuria scabra* (Holothuroidea; Echinodermata). Marine Biology 160, 3087–3101.

[brv12799-bib-0166] Post, A. L. , Lavoie, C. , Domack, E. W. , Leventer, A. , Shevenell, A. & Fraser, A. D. (2017). Environmental drivers of benthic communities and habitat heterogeneity on an East Antarctic shelf. Antarctic Science 29, 17–32.

[brv12799-bib-0167] Powell, E. N. (1977). Particle size selection and sediment reworking in a funnel feeder, *Leptosynapta tenuis* (Holothuroidea, Synaptidae). Internationale Revue der gesamten Hydrobiologie und Hydrographie 62, 385–408.

[brv12799-bib-0168] Purcell, S. S. , Conand, C. , Uthicke, S. & Byrne, M. (2016). Ecological roles of exploited sea cucumbers. Oceanography and Marine Biology: An Annual Review 54, 367–386.

[brv12799-bib-0169] Purcell, S. S. , Gossuin, H. , Agudo, N. S. & WorldFish Center (2009). Status and Management of the Sea Cucumber Fishery of La Grande Terre, New Caledonia: Studies and Reviews. The Worldfish Center, Penang.

[brv12799-bib-0170] Raff, R. A. & Byrne, M. (2006). The active evolutionary lives of echinoderm larvae. Heredity 97, 244–252. 16850040 10.1038/sj.hdy.6800866

[brv12799-bib-0171] Rahman, M. A. & Yusoff, F. (2017). Sea cucumber fisheries: market potential, trade, utilization and challenges for expanding the production in the South‐East Asia. International Journal of Advances in Chemical Engineering and Biological Sciences 4, 26–30.

[brv12799-bib-0172] Rahman, M. A. , Yusoff, F. M. & Arshad, A. (2015). Sea cucumber fisheries: global status, culture, management and extinction risks. International Journal of Chemical, Environmental and Biological Sciences 3(4), 344–348.

[brv12799-bib-0173] Renzi, M. , Blašković, A. , Bernardi, G. & Russo, G. F. (2018). Plastic litter transfer from sediments towards marine trophic webs: a case study on holothurians. Marine Pollution Bulletin 135, 376–385. 30301049 10.1016/j.marpolbul.2018.07.038

[brv12799-bib-0174] Resueño, M. A. & Angara, E. V. (2020). Species distribution, diversity, and abundance of sea cucumbers in tropical intertidal zones of Aurora, Philippines. Open Journal of Ecology 10, 768–777.

[brv12799-bib-0175] Rex, M. , Etter, R. , Morris, J. , Crouse, J. , McClain, C. , Johnson, N. , Stuart, C. , Deming, J. , Thies, R. & Avery, R. (2006). Global bathymetric patterns of standing stock and body size in the deep‐sea benthos. Marine Ecology Progress Series 317, 1–8.

[brv12799-bib-0176] Rhoads, D. C. & Young, D. K. (1971). Animal‐sediment relations in Cape Cod Bay, Massachusetts II. Reworking by *Molpadia oolitica* (Holothuroidea). Marine Biology 11, 255–261.

[brv12799-bib-0177] Roberts, D. (1979). Deposit‐feeding mechanisms and resource partitioning in tropical holothurians. Journal of Experimental Marine Biology and Ecology 37, 43–56.

[brv12799-bib-0178] Roberts, D. , Billett, D. S. M. , McCartney, G. & Hayes, G. E. (1991). Procaryotes on the tentacles of deep‐sea holothurians: a novel form of dietary supplementation. Limnology and Oceanography 36, 1447–1451.

[brv12799-bib-0179] Roberts, D. & Bryce, C. (1982). Further observations on tentacular feeding mechanisms in holothurians. Journal of Experimental Marine Biology and Ecology 59, 151–163.

[brv12799-bib-0180] Roberts, D. , Gebruk, A. , Levin, V. & Manship, B. A. D. (2000). Feeding and digestive strategies in deposit‐feeding holothurians. Oceanography and Marine Biology: An Annual Review 38, 257–310.

[brv12799-bib-0181] Roberts, D. & Moore, H. M. (1997). Tentacular diversity in deep‐sea deposit‐feeding holothurians: implications for biodiversity in the deep sea. Biodiversity and Conservation 6, 1487–1505.

[brv12799-bib-0182] Roberts, D. , Moore, H. M. , Berges, J. , Patching, J. W. , Carton, M. W. & Eardly, D. F. (2001). Sediment distribution, hydrolytic enzyme profiles and bacterial activities in the guts of *Oneirophanta mutabilis*, *Psychropotes longicauda* and *Pseudostichopus villosus*: what do they tell us about digestive strategies of abyssal holothurians? Progress in Oceanography 50, 443–458.

[brv12799-bib-0183] Ruhl, H. A. & Smith, K. L. (2004). Shifts in deep‐sea community structure linked to climate and food supply. Science 305, 513–515. 15273392 10.1126/science.1099759

[brv12799-bib-0184] Schulte, E. & Riehl, R. (1976). Electron microscope studies on the tentacles of *Lanice conchilega* (Polychaeta Sedentaria). Helgoländer Wissenschaftliche Meeresuntersuchungen 28, 191–205.

[brv12799-bib-0185] Sha, Y. , Liu, M. , Wang, B. , Jiang, K. , Sun, G. & Wang, L. (2016). Gut bacterial diversity of farmed sea cucumbers *Apostichopus japonicus* with different growth rates. Microbiology 85, 109–115.

[brv12799-bib-0186] Shiell, G. R. & Uthicke, S. (2005). Reproduction of the commercial sea cucumber *Holothuria whitmaei* [Holothuroidea: Aspidochirotida] in the Indian and Pacific Ocean regions of Australia. Marine Biology 148, 973–986.

[brv12799-bib-0187] Shimeta, J. (1996). Particle‐size selection by *Pseudopolydora paucibranchiata* (Polychaeta: Spionidae) in suspension feeding and in deposit feeding: influences of ontogeny and flow speed. Marine Biology 126, 479–488.

[brv12799-bib-0188] Sibuet, M. (1985). Quantitative distribution of echinoderms (Holothuroidea, Asteroidea, Ophiouroidea, Echinoidea) in relation to organic matter in the sediment, in deep sea basins of the Atlantic Ocean. In: Keegan, B.F., O'Connor, B.D.S. (Eds.), *Echinodermata. Proceedings of the Fifth International Echinoderm Conference*, 24–29 September 1984, Galway, Ireland. Balkema, Rotterdam, Boston, pp. 99–108.

[brv12799-bib-0189] Skewes, T. , Kinch, J. , Polon, P. , Dennis, D. , Seeto, P. , Taranto, T. , Lokani, P. , Wassenberg, T. , Koutsoukos, A. & Sarke, J. (2002). Research for sustainable use of Bêche‐de‐mer resources in Milne Bay Province, Papua New Guinea. CSIRO Final Report, 37.

[brv12799-bib-0190] Skewes, T. , Taylor, S. , Dennis, D. , Haywood, M. & Donovan, A. (2006). Sustainability assessment of the Torres Strait Sea cucumber fishery. CSIRO Final Report, 44.

[brv12799-bib-0191] Slater, M. J. , Jeffs, A. G. & Sewell, M. A. (2011). Organically selective movement and deposit‐feeding in juvenile sea cucumber, *Australostichopus mollis* determined in situ and in the laboratory. Journal of Experimental Marine Biology and Ecology 409, 315–323.

[brv12799-bib-0192] Sloan, N. A. & von Bodungen, B. (1980). Distribution and feeding of the sea cucumber *Isostichopus badionotus* in relation to shelter and sediment criteria of the Bermuda platform. Marine Ecology Progress Series 2, 257–264.

[brv12799-bib-0193] Smith, T. B. (1983). Tentacular ultrastructure and feeding behaviour of *Neopentadactyla mixta* (Holothuroidea: Dendrochirota). Journal of the Marine Biological Association of the United Kingdom 63, 301–311.

[brv12799-bib-0194] Sokolova, M. N. (1958). Feeding of deep sea bottom invertebrate deposit‐feeders. Transactions of the Institute of Oceanography 27, 123–153.

[brv12799-bib-0195] Sonnenholzner, J. (2003). Seasonal variation in the food composition of *Holothuria theeli* (Holothuroidea: Aspidochirotida) with observations on density and distribution patterns at the central coast of Ecuador. Bulletin of Marine Science 73, 527–543.

[brv12799-bib-0196] Sorokin, Y. I. (1972). Bacteria as food for coral reef fauna. Oceanology 12, 169–177.

[brv12799-bib-0197] Suchanek, T. H. , Williams, S. L. , Ogden, J. C. , Hubbard, D. K. & Gill, I. P. (1985). Utilization of shallow‐water seagrass detritus by Caribbean deep‐sea macrofauna: S^13^C evidence. Deep Sea Research Part II: Topical Studies in Oceanography 32, 201–214.

[brv12799-bib-0198] Suzumura, M. , Miyajima, T. , Hata, H. , Umezawa, Y. , Kayanne, H. & Koike, I. (2002). Cycling of phosphorus maintains the production of microphytobenthic communities in carbonate sediments of a coral reef. Limnology and Oceanography 47, 771–781.

[brv12799-bib-0199] Taddéi, D. (2006). *Transfert de matière et d'énergie dans les sédiments d'un complexe récifal anthropisé (Ile de la Réunion*, *Océan Indien)*. PhD Thesis: University of La Réunion, France. Technologies.

[brv12799-bib-0200] Taghon, G. L. (1989). Modeling deposit feeding. In Ecology of Marine Deposit Feeders (eds G. Lopez , G. Taghon and J. Levinton ), pp. 223–246. Springer, New York.

[brv12799-bib-0201] Taghon, G. L. & Jumars, P. A. (1984). Variable ingestion rate and its role in optimal foraging behavior of marine deposit feeders. Ecology 65, 549–558.

[brv12799-bib-0202] Taylor, M. L. , Gwinnett, C. , Robinson, L. F. & Woodall, L. C. (2016). Plastic microfibre ingestion by deep‐sea organisms. Scientific Reports 6, 33997. 27687574 10.1038/srep33997 PMC5043174

[brv12799-bib-0203] Thiel, H. , Pfannkuche, O. , Schriever, G. , Lochte, K. , Gooday, A. J. , Hemleben, C. , Mantoura, R. F. C. , Turley, C. M. , Patching, J. W. & Riemann, F. (1989). Phytodetritus on the Deep‐Sea floor in a central oceanic region of the Northeast Atlantic. Biological Oceanography 6, 203–239.

[brv12799-bib-0204] Thurston, M. H. , Bett, B. J. , Rice, A. L. & Jackson, P. A. B. (1994). Variations in the invertebrate abyssal megafauna in the North Atlantic Ocean. Deep Sea Research Part I: Oceanographic Research Papers 41, 1321–1348.

[brv12799-bib-0205] Thurston, M. H. , Rice, A. L. & Bett, B. J. (1998). Latitudinal variation in invertebrate megafaunal abundance and biomass in the North Atlantic Ocean abyss. Deep Sea Research Part II: Topical Studies in Oceanography 45, 203–224.

[brv12799-bib-0206] Toral‐Granda, V. , Lovatelli, A. & Vasconcellos, M. (2008). Sea Cucumbers: A Global Review of Fisheries and Trade. FAO, Rome.

[brv12799-bib-0207] Trefz, M. (1958). *The Physiology of Digestion of Holothuria Atra Jager with Special Reference to its Role in the Ecology of Coral Reefs*. PhD Thesis: University of Hawaii.

[brv12799-bib-0208] Tyler, P. A. , Young, C. M. , Billett, D. S. M. & Giles, L. A. (1992). Pairing behaviour, reproduction and diet in the deep‐sea holothurian genus *Paroriza* (Holothurioidea: Synallactidae). Journal of the Marine Biological Association of the United Kingdom 72, 447–462.

[brv12799-bib-0209] Uthicke, S. (1994). Distribution patterns and growth of two reef flat holothurians, *Holothuria atra* and *Stichopus chloronotus* . In Echinoderms through Time: Proceedings of the 8th International Echinoderm Conference (eds B. David , A. Guille , J. P. Feral and M. Roux ), pp. 569–576. Dijon. A.A. Balkema, Rotterdam.

[brv12799-bib-0210] Uthicke, S. (1999). Sediment bioturbation and impact of feeding activity of *HolothurIa* (*Halodeima*) *atra* and *Stichopus chloronotus*, two sediMent feeding holothurians, at Lizard Island, great barrier reef. Bulletin of Marine Science 64, 129–141.

[brv12799-bib-0211] Uthicke, S. & Karez, R. (1999). Sediment patch selectivity in tropical sea cucumbers (Holothurioidea: Aspidochirotida) analysed with multiple choice experiments. Journal of Experimental Marine Biology and Ecology 236, 69–87.

[brv12799-bib-0212] Uthicke, S. & Klumpp, D. (1998). Microphytobenthos community production at a near‐shore coral reef: seasonal variation and response to ammonium recycled by holothurians. Marine Ecology Progress Series 169, 1–11.

[brv12799-bib-0213] Van Cauwenberghe, L. , Vanreusel, A. , Mees, J. & Janssen, C. R. (2013). Microplastic pollution in deep‐sea sediments. Environmental Pollution 182, 495–499. 24035457 10.1016/j.envpol.2013.08.013

[brv12799-bib-0214] Van Iperen, J. M. , Van Weering, T. C. E. , Jansen, J. H. F. & van Bennekom, A. J. (1987). Diatoms in surface sediments of the Zaire deep‐sea fan (se Atlantic Ocean) and their relation to overlying water masses. Netherlands Journal of Sea Research 21, 203–217.

[brv12799-bib-0215] Wang, F. , Yang, H. , Gabr, H. R. & Gao, F. (2008). Immune condition of *Apostichopus japonicus* during aestivation. Aquaculture 285, 238–243.

[brv12799-bib-0216] Ward‐Rainey, N. , Rainey, F. A. & Stackebrandt, E. (1996). A study of the bacterial flora associated with *Holothuria atra* . Journal of Experimental Marine Biology and Ecology 203, 11–26.

[brv12799-bib-0217] Whitlatch, R. B. & Obrebski, S. (1980). Feeding selectivity and coexistence in two deposit‐feeding gastropods. Marine Biology 58, 219–225.

[brv12799-bib-0218] Wiebe, P. H. , Boyd, S. H. & Winget, C. (1976). Particulate Matter Sinking to the Deep‐Sea Floor at 2000 M in the Tongue of the Ocean, Bahamas, with a Description of a New Sedimentation Trap. Woods Hole Oceanographic Institution, Woods Hole, MA.

[brv12799-bib-0219] Wiedemeyer, W. L. (1994). Biology of small juveniles of the tropical holothurian *Actinopyga echinites*: growth, mortality, and habitat preferences. Marine Biology 120, 81–93.

[brv12799-bib-0220] Wigham, B. D. , Galley, E. A. , Smith, C. R. & Tyler, P. A. (2008). Inter‐annual variability and potential for selectivity in the diets of deep‐water Antarctic echinoderms. Deep Sea Research Part II: Tropical Studies in Oceanography 55, 2478–2490.

[brv12799-bib-0221] Wigham, B. D. , Hudson, I. R. , Billett, D. S. M. & Wolff, G. A. (2003). Is long‐term change in the abyssal Northeast Atlantic driven by qualitative changes in export flux? Evidence from selective feeding in deep‐sea holothurians. Progress in Oceanography 59, 409–441.

[brv12799-bib-0222] Witbaard, R. , Duineveld, G. C. A. , Kok, A. , van der Weele, J. & Berghuis, E. M. (2001). The response of *Oneirophanta mutabilis* (Holothuroidea) to the seasonal deposition of phytopigments at the porcupine abyssal plain in the Northeast Atlantic. Progress in Oceanography 50, 423–441.

[brv12799-bib-0223] Wolcott, T. G. (1981). Inhaling without ribs: the problem of suction in soft‐bodied invertebrates. The Biological Bulletin 160, 189–197.

[brv12799-bib-0224] Wolfe, K. & Davey, M. (2020). Localised high‐density population of a sea cucumber on a Malaysian coral reef. Coral Reefs 39, 33–38.

[brv12799-bib-0225] Wood, E. J. F. (1956). Diatoms in the ocean deeps. Pacific Science 10, 377–381.

[brv12799-bib-0226] Woodall, L. C. , Sanchez‐Vidal, A. , Canals, M. , Paterson, G. L. J. , Coppock, R. , Sleight, V. , Calafat, A. , Rogers, A. D. , Narayanaswamy, B. E. & Thompson, R. C. (2014). The deep sea is a major sink for microplastic debris. Royal Society Open Science 1, 140317. 26064573 10.1098/rsos.140317 PMC4448771

[brv12799-bib-0227] WoRMS Editorial Board (2020). World Register of Marine Species. Electronic file available at http://www.marinespecies.org at VLIZ. Accessed 24.10. 2020

[brv12799-bib-0228] Yamanouchi, T. (1939). Ecological and physiological studies on the holothurians in the coral reef of Palao Island. Palao Tropical Biology Studies 25, 603–634.

[brv12799-bib-0229] Yamanouchi, T. (1956). The daily activity rhythms of the holothurians in the coral reef of Palao Islands. Seto Marine Biological Laboratory 5, 347–362.

[brv12799-bib-0230] Yang, H. , Yuan, X. , Zhou, Y. , Mao, Y. , Zhang, T. & Liu, Y. (2005). Effects of body size and water temperature on food consumption and growth in the sea cucumber *Apostichopus japonicus* (Selenka) with special reference to aestivation. Aquaculture Research 36, 1085–1092.

[brv12799-bib-0231] Yingst, J. Y. (1976). The utilization of organic matter in shallow marine sediments by an epibenthic deposit‐feeding holothurian. Journal of Experimental Marine Biology and Ecology 23, 55–69.

[brv12799-bib-0232] Yingst, J. Y. (1982). Factors influencing rates of sediment ingestion by *Parastichopus parvimensis* (Clark), an epibenthic deposit‐feeding holothurian. Estuarine, Coastal and Shelf Science 14, 119–134.

[brv12799-bib-0233] Young, C. M. & Chia, F.‐S. (1982). Factors controlling spatial distribution of the sea cucumber *Psolus chitonoides*: settling and post‐settling behavior. Marine Biology 69, 195–205.

[brv12799-bib-0234] Yuan, X. , Yang, H. , Wang, L. , Zhou, Y. , Zhang, T. & Liu, Y. (2007). Effects of aestivation on the energy budget of sea cucumber *Apostichopus japonicus* (Selenka) (Echinodermata: Holothuroidea). Acta Ecologica Sinica 27, 3155–3161.

[brv12799-bib-0235] Zhang, X. , Nakahara, T. , Miyazaki, M. , Nogi, Y. , Taniyama, S. , Arakawa, O. , Inoue, T. & Kudo, T. (2012). Diversity and function of aerobic culturable bacteria in the intestine of the sea cucumber *Holothuria leucospilota* . The Journal of General and Applied Microbiology 58, 447–456. 23337580 10.2323/jgam.58.447

[brv12799-bib-0236] Zobell, C.E. & Morita, R.Y. (1959). Deep‐sea bacteria. Galathea Report, Copenhagen 1, 139–154.

